# Translational validity of quantitative sensory testing in chronic pain neuro-sensitization: guide of use and interpretation in osteoarthritis animal models

**DOI:** 10.3389/fpain.2025.1709275

**Published:** 2025-12-10

**Authors:** Marilyn Frézier, Aliénor Delsart, Manuela Lefort-Holguin, Sarah Lachapelle, Colombe Otis, Bertrand Lussier, Hélène Beaudry, Aude Castel, Eric Troncy

**Affiliations:** 1 Groupe de Recherche en Pharmacologie Animale du Québec (GREPAQ), Université de Montréal, St.-Hyacinthe, QC, Canada; 2 Osteoarthritis Research Unit, University of Montreal Hospital Research Center (CRCHUM), Montréal, QC, Canada; 3 Department of Clinical Sciences, Faculty of Veterinary Medicine, Université de Montréal, St.-Hyacinthe, QC, Canada

**Keywords:** musculoskeletal, conditioned pain modulation, inhibition, temporal summation, facilitation, rodent, feline, canine

## Abstract

Chronic osteoarthritis (OA) pain is a complex nociplastic condition that affects humans, as well as cats and dogs. This review summarizes the physiology of pain in healthy individuals, the physiopathology of OA pain, and the use of quantitative sensory testing (QST) to objectively assess somatosensory sensitization associated with chronic OA pain. It discusses the translation of human OA pain phenotype profiles to animals, the management of neuro-sensitization with currently prescribed treatments, and complementary methods for evaluating neuro-sensitization, such as electrodiagnostic testing. Additionally, this review serves as a practical guide for standardizing QST in rats, cats, and dogs, with explanatory appendices. It was hypothesized that in translational comparison with the human condition, OA-induced rat models and naturally occurring OA in cats and dogs would exhibit similar somatosensory sensitization profiles. As observed in human OA, an imbalance between facilitatory and inhibitory endogenous controls is also evident in animal OA. This dysregulation can be characterized using QST and underlies the distinct nociceptive phenotypes. Confirming and validating OA pain profiles will promote a patient-tailored approach to effectively alleviate neuro-sensitization in humans and animals.

## 1 Introduction

Osteoarthritis (OA) is one of the most common degenerative diseases affecting both humans and companion animals, with a prevalence of approximately 15%–25% in adults, increasing exponentially with age (1–3). It involves joint mobility loss, functional impairment and the development of chronic pain, significantly reducing patients' quality of life (4). Initially, this pathology was defined as an inflammatory disease centered on cartilage degeneration, bone remodeling, synovial inflammation and soft-tissue alterations (1). Increasing evidence supports the notion that OA is not restricted to the joint but may present systemic manifestations, particularly affecting the nervous system. Indeed, chronic OA pain is characterized by low-grade inflammatory activation, which contributes to neuro-sensitization and neuroplasticity, ultimately establishing a nociplastic pain profile (Table 1 for type of pain definitions) (5). The complexity of chronic OA pain makes it difficult to diagnose and manage both in humans and animals (2, 3, 5). Alterations in pain sensory pathways contribute to this complexity, resulting in either a loss or gain of sensory function (i.e., hypo- or hyper-sensitivity) (6). Quantitative sensory testing (QST) provides objective quantification of somatosensory sensitivity and was described in healthy and OA populations, including humans, rat models, cats and dogs (7–11). In humans, a multitude of OA pain phenotypes were described using QST, allowing personalized patient care (12–14). Despite the promising potential of QST for characterizing OA pain in animals, a lack of standardization has been reported (10, 11) and few studies have been conducted. To address this gap in the literature, the *Groupe de recherche en pharmacologie animale du Québec* (GREPAQ) developed a standardized panel of reliable QST outcomes to characterize chronic OA pain in induced rat models (15, 16) and in pets (8, 11), thereby contributing to the translational research in humans (17). These protocols are presented in appendices.

**Table 1:** Pain type definitions based on the international association for the study of pain and relevant articles (36, 187–189).

Type of pain	Definition
Acute	Caused by tissue damage (e.g., illness, injury, or medical/surgical traumatic procedures) Serves a protective role by signaling actual or potential tissue injury and promoting avoidance or healing behaviors Generally short-lived (<3 months) Resolves when the damage is healed Classed as an appropriate pain
Chronic	Can stem from an ongoing health condition that cannot be cured and can manifest in a transient, variable, or constant manner Persists beyond the usual recovery time from an injury or illness (>3 months) Can be appropriate, but often evolves as a maladaptive process where pain has lost its protective role and becomes a disease in itself (pathological pain) Can be characterized as inflammatory, neuropathic or nociplastic pain
Nociceptive	Arises from actual or threatened damage to non-neural tissue Activates nociceptors Most often an appropriate pain
Inflammatory	Happens when the immune system activates in response to tissue injury or infection In addition to redness or swelling, exacerbates sensitivity to feelings of pain Acute inflammation: normal protective process against cellular aggression that also promotes repair and recovery Persistent (micro) inflammation: can lead to neuropathic and/or nociplastic pain
Neuropathic	Direct consequence of a damage or disease/dysfunction affecting the somatosensory nervous system From injuries or illness that affect the spinal cord and brain (e.g., disc hernia) or the peripheral nervous system Leads to neural dysfunction Causes persistent or exaggerated maladaptive pain responses, often described to burning, shooting, or stabbing
Nociplastic	Results from altered nociceptive processing in the nervous system Can occur without clear evidence of tissue injury or somatosensory system damage. Involves maladaptive peripheral and central neuro-sensitization that amplifies pain signals, causing abnormal responses such as allodynia and hyperalgesia Commonly seen in chronic pain degenerative conditions like osteoarthritis, where pain persists despite limited structural damage.

This scoping review summarizes the physiology of pain in healthy individuals, the physiopathology of OA pain and the QST outcomes currently used to characterize the somatosensory alterations associated with OA in humans and animals (rats, cats and dogs). The review was conducted using a comprehensive search strategy in PubMed and Google Scholar, with full search scripts provided in the Supplementary Material (Table S1). As no studies focusing on temporomandibular and facet joint OA in cats or dogs have yet investigated neuro-sensitization, these conditions were not included in this review. The research hypothesis was translational in nature, positing that neuro-sensitization phenotypes would be similar in OA-induced rat models as well as in cats and dogs with naturally occurring OA. The first aim of this review was to provide a comprehensive and translational overview of QST methods used to assess somatosensory alterations associated with OA pain in rats, cats and dogs. The second aim was to propose a practical framework for constructing chronic OA pain sensitization profiles across these species, thereby supporting the development of personalized medicine approaches through the selection of tailored treatment strategies based on each patient's specific sensitization phenotype.

## 2 Physiology of pain

**Figure 1:**
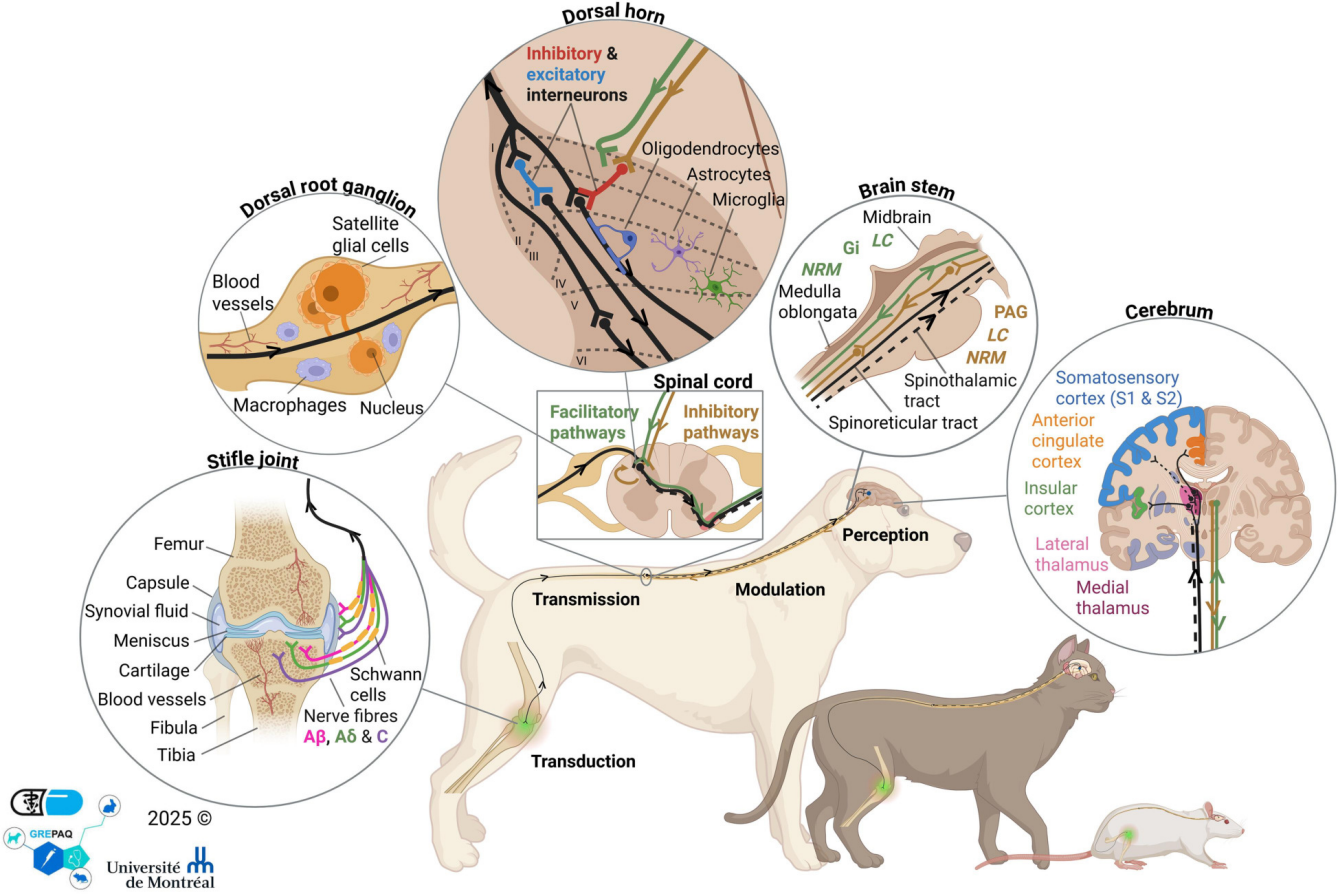
Overview of the normal pain pathways (transduction, transmission, perception and modulation) from the stifle joint to the brain, via the dorsal root ganglion, spinal cord dorsal horn and brain stem in the healthy state. PAG, Periaqueductal grey; NRM, Nucleus raphe magnus; LC, Locus coeruleus; Gi, Gigantocellular reticular nucleus. Created in BioRender. Frezier, M. (2025) https://BioRender.com/85ftir7.

Mammals share a closely related system of detection, quantification and most importantly, control of nociceptive signals (Figure 1). Nociception refers to the processing of noxious stimuli (e.g., chemical, thermal, or mechanical) by the peripheral (PNS) and central nervous systems (CNS), associated with (potential) tissue injury. Therefore, nociceptor activation initiates the transduction, transmission, integration and perception of pain signals, which are simultaneously modulated at all levels of the nociceptive pathways to create a subjective experience of pain (18). Pain modulation is endogenously regulated by local structures (e.g., spinal interneurons) and/or higher-order brain centers and creates a dynamic balance between facilitation and inhibition (I/F) of pain (19, 20) to provide the most adapted nociceptive response. Pain represents a complex and highly individualized experience, shaped by physiological and psychological states that can alter the balance and effectiveness of modulatory pathways.

Pain sensation often begins with the activation of transient receptor potential channels located on the free nerve endings of nociceptors (18). The transduction of noxious stimuli initiates the genesis of an action potential (AP), which is transmitted along the axons of first-order neurons (18). This signal propagates to neuronal cell bodies located in the dorsal root ganglion (DRG) for stimuli originating from the body or the trigeminal ganglia for stimuli from the face, before reaching the spinal cord (18, 20). There are two primary nociceptors corresponding to first-order neurons: Aδ- and C-fibers. The Aδ-fibers are myelinated with a medium axon diameter (1–5 μm) and are responsible for the rapid transmission of thermal and mechanical pain signals, functioning as an early warning system (Figure 1). In contrast, the C-fibers are unmyelinated, have a smaller diameter (0.02–1.5 μm), and are polymodal, enabling them to relay pain intensity and contribute to prolonged pain perception. These fibers carry approximately 70% of nociceptive afferent input entering the spinal cord. Both nociceptive fiber types differ from a third type of primary afferent fiber: Aβ-fibers. These myelinated fibers have a larger diameter (6–12 μm) and rapidly conduct non-noxious mechanical stimuli, such as touch and vibration. Primary afferent fibers synapse with second-order neurons or interneurons in the Rexed laminae of the spinal dorsal horn: Aδ nociceptors project to laminae I and V; C nociceptors to laminae I and II and Aβ afferents to laminae III—IV (18, 20). Despite their specific distribution, primary afferent fibers can penetrate other laminae through collateral branches. Second-order neurons are classified into three types: low-threshold neurons that respond to innocuous stimuli, nociceptive-specific neurons that respond to high-threshold noxious stimuli, and wide dynamic range (WDR) neurons (21). Spinal WDR neurons possess large receptive fields and integrate all types of peripheral sensory input, both innocuous and nociceptive, from Aβ-, Aδ-, and C-fibers (21). Nociceptor activation leads to the release of different neurotransmitters [e.g., inflammatory mediators, glutamate, glycine, γ-aminobutyric acid (GABA)] and neuropeptides (e.g., substance P, calcitonin gene-related peptide, somatostatin, enkephalins), which activate ascending pain pathways such as the lateral spinothalamic tract or the spinoreticular tract in a controlled manner (18, 20). Projection neurons within these tracts synapse with third-order neurons in the thalamic nuclei, specifically in the ventrobasal or centromedian complexes, which serve as a relay and integrative hubs for nociceptive information. From there, nociceptive signals are transmitted to higher brain centers involved in the perception, integration, and emotional processing of pain. The lateral spinothalamic tract projects to the primary (S1) and secondary (S2) somatosensory cortices, where the localization, intensity, and temporal characteristics of the stimulus are encoded, defining the sensory-discriminative component of pain. In contrast, the medial spinoreticular tract projects to the midbrain reticular formation, periaqueductal gray (PAG), and limbic areas (cingulate gyrus and insular cortex), contributing to the affective-motivational component of pain. The limbic system, including the amygdala, anterior cingulate cortex, and insula, integrates nociceptive input with emotional and behavioral responses, linking sensory processing to affective experience. The PAG and associated pontine structures play a pivotal role in integrating ascending nociceptive input with descending modulatory systems. Together, these supraspinal regions transform nociceptive input into a multidimensional experience that encompasses sensory, affective, and cognitive components of pain (18, 20).

The nervous system can suppress or amplify nociceptive signals at different levels along the pain pathway (19). At the supraspinal level, descending inhibitory and facilitatory modulatory pathways form a complex circuit involving various brain structures (Figure 1). The endogenous opioid pathway, the primary analgesic system, originates mainly from the PAG and, to a lesser extent, from the rostral ventral medulla (RVM). It releases enkephalins, endorphins, and dynorphins into the dorsal horn of the spinal cord (22). Similarly, the endocannabinoid system appears to have an inhibitory effect on the pain pathway via the activation of two cannabinoid receptors: CB1R and CB2R (23). Additionally, the action of noradrenergic (i.e., norepinephrine) and serotoninergic pathways, originating from the *locus coeruleus* and medulla (*nucleus raphe magnus,* gigantocellular reticular nucleus and RVM) respectively, can result in inhibition or facilitation (20, 24). Dopaminergic pathways, originating from hypothalamus, also appear to play a role in pain modulation through either inhibitory or facilitatory effects (20).

Supraspinal structures such as the RVM can modulate pain perception by activating inhibitory (GABAergic and glycinergic) or excitatory (glutaminergic) interneurons at the spinal cord level (25). According to the gate control theory of pain proposed by Melzack and Wall in 1965, activation of Aβ-fibers by non-noxious stimuli recruits inhibitory interneurons in the dorsal horn, thereby blocking pain signals transmitted by nociceptive Aδ- and C-fibers (26). Recently, it has been shown that pain signaling can be modulated by glial and immune cells of PNS and CNS (27). In the physiology of pain, these suppressive or amplifying control systems keep the I/F balance in equilibrium.

## 3 From appropriate to maladaptive pain in osteoarthritis

**Figure 2:**
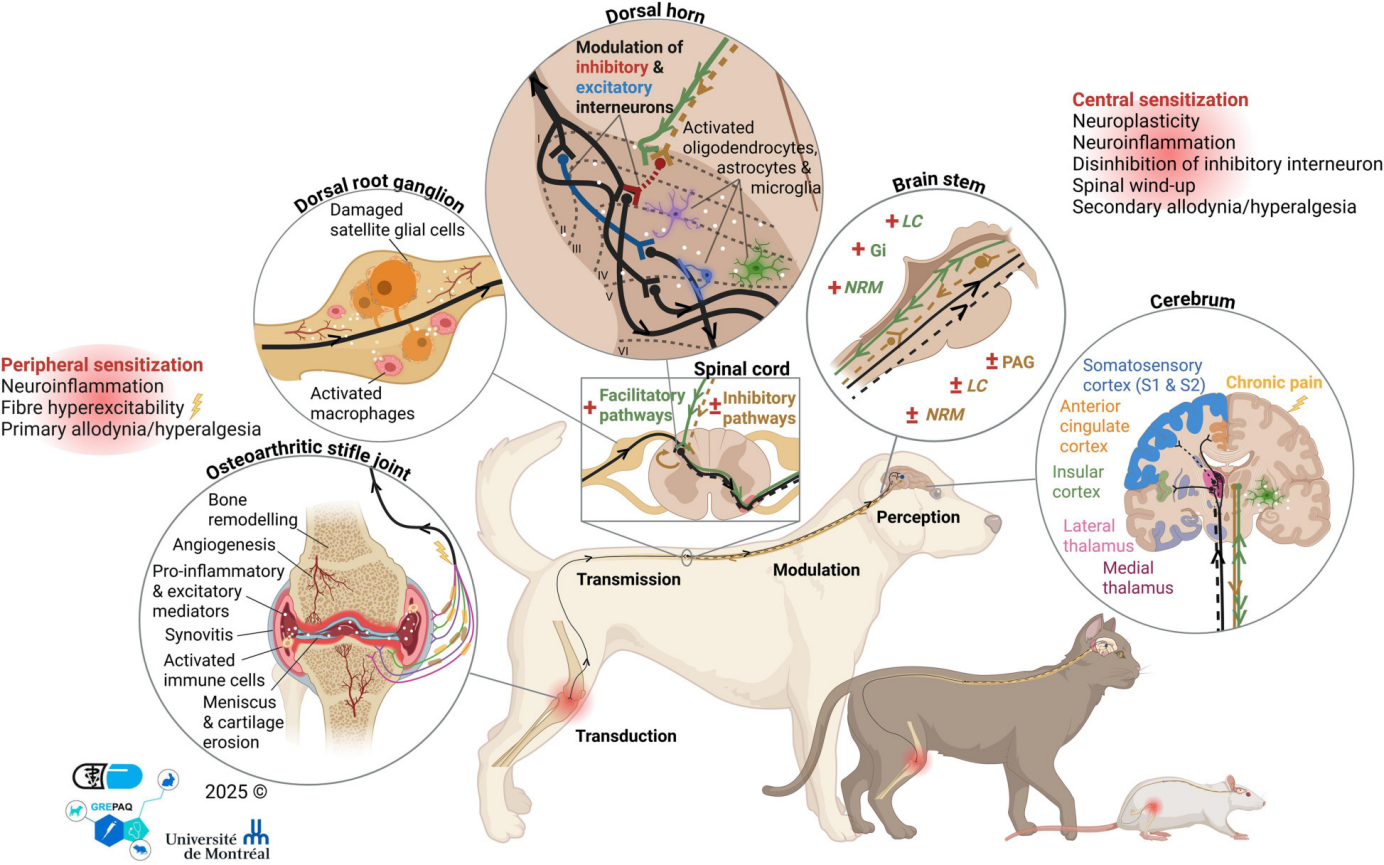
Illustration of the alterations associated with chronic stifle osteoarthritic pain leading to peripheral and central sensitization, and of the dysfunction of modulation systems (facilitatory and inhibitory pathways) from transduction to perception. PAG, Periaqueductal grey; NRM, Nucleus raphe magnus; LC, Locus coeruleus, Gi, Gigantocellular reticular nucleus. Created in BioRender. Frezier, M. (2025) https://BioRender.com/85ftir7.

Chronic pain is associated with neurochemical changes that can lead to PNS and CNS neuro-sensitization. The latter modulates pain perception at the somatosensory cortices (28, 29). In patients with chronic pain, particularly OA (Figure 2), the nociceptive system undergoes transformation through the activation of immune-inflammatory pathways, enhanced sensitivity of primary afferent nociceptors, neuroplasticity, excessive neuropathic excitability, and/or an imbalance between facilitatory pain processes and endogenous inhibitory controls (i.e., maladaptive pain modulation) (29). When cells damage occurs (e.g., through cartilage degradation), immune cells are activated, and pro-inflammatory and excitatory mediators are released locally (27). Some of these mediators consist of pro-inflammatory cytokines, prostaglandins, matrix metalloproteinases, alpha tumor necrosis factor, nerve growth factor (NGF), neuropeptides, etc. (27). Acting synergistically, these mediators form a “sensitizing (inflammatory) soup”, leading to the peripheral sensitization of surrounding nociceptors (27). Indeed, nociceptors can themselves promote peripheral sensitization through neurogenic inflammation. As an example, an elevated NGF concentration was reported in the chondrocytes of OA humans (30) and the synovial fluid of OA dogs with chronic (but not acute) lameness (31). The NGF binds to its receptor TrkA, which is located on primary afferent fibers and immune cells. Following internalization, the NGF/TrkA complex is transported to the cell body in the DRG, where it modulates the expression of receptors involved in nociception, as well as pro-nociceptive neurotransmitters (e.g., substance P and calcitonin gene-related peptide), ultimately leading to neuro-sensitization (32). By secreting pro-inflammatory and excitatory mediators (mainly neuropeptides), nociceptors antidromically activate neighboring nerve endings via DRG signaling, further attracting immune cells and perpetuating a vicious circle (27). This neuroinflammatory phenomenon, in conjunction with neuronal plasticity and hyperexcitability, contributes to peripheral sensitization (33). Neuronal plasticity is observed with increasing innervation of the affected joint and angiogenesis, both promoted by NGF and vascular endothelial growth factor (34, 35); while hyperexcitability is marked by the release of pro-inflammatory substances triggering receptors activation and overexpression of protein receptors involved in pain signalization (28, 33). Together, local inflammation, neuroinflammation, neuronal plasticity and hyperexcitability lower the threshold for Aδ- and C-fiber activation (27). The threshold decreases and the increase in firing rates often manifest themselves through allodynia and hyperalgesia, two characteristic phenomena of maladaptive pain and central sensitization. According to the International Association for the Study of Pain (IASP), the clinical term “allodynia” refers to the perception of pain following a stimulus that does not normally provoke pain (i.e., light touch), whereas “hyperalgesia” denotes an exaggerated pain perception following a suprathreshold stimulus (36).

The first evidence for a central component in acute pain hypersensitivity was provided in 1983 (37), and the term central sensitization was coined by Woolf and King (38) in 1989 after studies in rats showed that neurons in the spinal cord become hyperexcitable over time after injury. Central sensitization can be maintained with or without continued peripheral input, and chemical, structural, and functional changes in the CNS may ultimately lead to a persistent, heightened state of neural reactivity (39). The original description of central sensitization referred to an activity- or use-dependent form of functional synaptic plasticity that resulted in pain hypersensitivity after an intense noxious stimulus. Increased nociceptive inputs associated with OA sensitization contributes to CNS modification, promoting the development of chronic pain (28, 40). Central sensitization is characterized by microglial activation, neuronal hyperexcitability (with a key involvement of glutamate and its receptors), and plasticity, occurring both in the dorsal horn and in cerebral structures (33, 39). Within the Rexed laminae, neuroplasticity and hyperexcitability can lead to confusion of innocuous signals and/or the amplification of nociceptive input by WDR neurons, resulting in primary (local) and secondary (widespread) allodynia and hyperalgesia (39). Additionally, disinhibition of inhibitory interneurons and activation of excitatory interneurons disturb the I/F balance, further exacerbating central sensitization development and pathological pain (25). In experimental OA models in rats (15, 16, 41–45), and surgically induced OA models in dogs (46), central sensitization results in increased spinal neuropeptides (mainly substance P, calcitonin gene-related peptide and bradykinin) in the synaptic cleft, enhancing release of neurotrophic factors such as brain-derived neurotrophic factor, and increased responsiveness and recruitment of novel inputs (Aβ-fiber). Spinal sensitization is the long-term (persistent) result of two activity-dependent and short- (minutes) or long-lasting (hours), transient phenomena: wind-up and homosynaptic potentiation respectively. These correspond to the early phosphorylation-dependent phase, mainly associated to rapid changes in glutamate receptor and ion channel properties, and to the later, longer-lasting, transcription-dependent phase, driving synthesis of new proteins responsible for the longer-lasting form of central sensitization observed in several pathological conditions (47). The wind-up phenomenon refers to the summation of AP initiated by low-frequency trains of innocuous or noxious stimuli at C-fibers (48). Seconds of peripheral activation can result in minutes of post-synaptic depolarization in the spinal dorsal horn, facilitating pain signals transmission and intensity, correlating with increased pain perception (temporal summation) (48, 49). The recruitment of C-fibers can also lead to homosynaptic potentiation, phenomenon that can sustain facilitation for several hours (39). The opening of post-synaptic receptors enables spatial summation of AP through the activation and recruitment of silent C-fibers, extending hyperalgesia beyond the primary site of pain (secondary hyperalgesia) (50). In addition to stimulus-evoked AP, spontaneous firing of WDR neurons can occur, further contributing to central sensitization (21). At the cerebral level, OA-induced structural and functional neuroplastic changes remain poorly described and sometimes contradictory. Systematic reviews of neuro-imaging studies did not significantly reveal structural changes. However, they suggested that OA patients exhibit increased activation of the somatosensory cortices (S1 and S2) and may have reduce volume activity in anterior insula, and reduced gray matter volume, associated with sensitization parameters (51, 52). In rats with advanced OA pain, increased activity in PAG was observed, particularly with aging, potentially contributing to pain chronicity (53). In OA cats, positron emission tomography imaging revealed increased brain metabolism in the S2 cortex, reflecting the sustained nociceptive inputs, as well as in the thalamus and PAG, demonstrating involvement of inhibitory pathways compared to healthy cats (54).

Alterations in peripheral and central pain-related (sensory) nociceptive pathways can lead to an imbalance in I/F pain modulation mechanisms (29, 39, 55, 56). This I/F imbalance illustrates a phenomenon called “loss/gain” pain. The term “loss” refers to functional sensory deficit or activation of endogenous inhibitory pain control (i.e., loss corresponding to an increased pain threshold) (6). Conversely, the term “gain” denotes increased sensitization, facilitation of pain signals, and/or impaired endogenous inhibitory pain control processes through inactivity or fatigue (i.e., gain corresponding to decreased pain thresholds) (6). As previously discussed, glutamatergic, neuropeptidergic, opioid, serotoninergic, noradrenergic and cannabinoid pathways play complex roles in pain modulation. The adaptive serotoninergic system can shift between antinociceptive (inhibitory) and pronociceptive (facilitatory) roles depending on receptor activation within the spinal cord (57). In chronic OA pain, these pathways become altered and shift towards pain facilitation. Notably, increased serotoninergic facilitatory drive via 5-HT3 receptors was reported in the spinal cord during later stages of the monosodium iodoacetate (MIA) OA rat model (57). Additionally, evidence of an increase in spinal inflammatory and tachykinins neuropeptides (16, 41, 43) along with diminished inhibitory noradrenergic (57) and cannabinoid (58) descending pathways have been observed in MIA rats, further contributing to the I/F imbalance. Similar findings were reported in a surgically induced OA model in rats (15, 42, 44, 45).

## 4 Quantitative sensory testing to assess osteoarthritis pain mechanisms

*Quantitative sensory testing enables the characterization of somatosensory changes* (i.e., gain or loss of sensory function), reflecting peripheral and/or central sensitization as well as modulation by endogenous control mechanisms, using a non-invasive panel of methods (Table 2) (6, 59, 60). Given increasing evidence of I/F imbalance in OA pain, QST protocols must incorporate and evaluate this dimension to avoid misinterpretation and mismanagement (5).

**Table 2:** Summary of QST used and validated in human clinics and in veterinary medicine to evaluate OA pain.

QST	Pain profile	Fibers or pathways assessed	Equipment	Validation and reliability
Neuro-sensitization				
Thermal detection threshold	Thermal hypo- or hyperalgesia	Aδ and C	Thermode or thermal plate (cold or warm)	- Humans: specific, sensitive, repeatable and reproducible (7, 10, 12, 61) - Rats: specific and sensitive but less reliable than mechanical (9) - Cats: not enough studies to conclude (8) - Dogs: conflictual results on specificity and repeatability (11)
Punctate pain threshold (PcPT)/Paw withdrawal threshold (PWT)	Mechanical static hypoesthesia or allodynia	Aβ	von Frey® filaments or electronic esthesiometer	- Humans: specific, sensitive and repeatable (62, 63) - Rats: specific, sensitive, repeatable and reproducible (9, 15, 16, 41–45, 107) - Cats: specific, sensitive, repeatable and reproducible (8) - Dogs: sensitive but conflictual results on specificity and repeatability (11, 66)
Tactile sensitization	Mechanical dynamic hypoesthesia or allodynia	Aβ	Soft brush, cotton swab, Q-tip	- Humans: specific and sensitive (77) - Rats: specific and sensitive in anesthetized rats (74–76) - Cats: N/A - Dogs: specific, sensitive and repeatable [dogs with osteosarcoma (67)].
Pressure pain threshold (PPT)	Mechanical hypo- or hyper-algesia	Aδ and C	Pressure algometer	- Humans: specific, sensitive, repeatable and reproducible (7, 10, 12, 61) - Rats: specific, sensitive, repeatable and reproducible (69–71) - Cats: N/A - Dogs: sensitive but conflictual results on specificity and repeatability (11)
Temporal summation (TS)	Central sensitization—Spinal hyperexcitability (wind-up)	- Aβ and C - WDR neurons	Standardized repeated stimuli (thermal, pin prick, pressure or electrical)	- Humans: specific, sensitive, repeatable and reproducible (10, 12, 61, 109) - Rats: specific (16, 84) - Cats: specific and sensitive (8, 91) - Dogs: specific (85)
Pain modulation				
Conditioned pain modulation (CPM)	Endogenous inhibition—Diffuse noxious inhibitory control (DNIC)	- Aδ and C - Noradrenergic, serotoninergic and opioid pathways - Inhibitory interneurons	Conditioning stimulus (chemical, ischemic, mechanical or thermal) Pre/post PWT or PPT	- Humans: specific, sensitive, mitigated reliability (12, 61, 89) - Rats: specific, sensitive and repeatable in anesthetized (57, 90) or conscious animals (16, 84, 107) - Cats: specific (91) - Dogs: specific [anesthetized (92, 93)] and repeatable [ (85); osteosarcoma (67)]
Temporal summation (TS)	Endogenous facilitation—Spinal hyperexcitability	- Aβ and C - WDR neurons - Serotoninergic and dopaminergic pathways - Excitatory interneurons	Nociceptive sensitivity measurement after TS (thermal or pin prick) Pre/post PWT or thermal detection threshold	- Humans: N/A - Rats: specific (84) - Cats: N/A - Dogs: N/A

### 4.1 Peripheral and central sensitization

#### 4.1.1 Peripheral sensitization—paw withdrawal and pressure thresholds

Peripheral sensitization, mostly related to inflammatory hypersensitivity, contributes to the sensitization of the nociceptive system (neuro-sensitization). It appears to play a major role in altered heat but not mechanical sensitivity, which is a major feature of central sensitization (39). “Peripheral sensitization” corresponding to specific QSTs, as defined in the remaining of the text, can be assessed by measuring mechanical or thermal sensitivity through recording thresholds or latency times. Mechanical paw withdrawal threshold (PWT) tests theoretically Aβ-fibers responsiveness, while thermal PWT and pressure pain threshold (PPT) assess Aδ- and C-fibers activity (60). Neuro-sensitization can occur at the primary site (i.e., at the local site of injury) or secondary site (i.e., remote to the affected area). As mentioned, increased sensitivity in the assessed fibers manifests as allodynia or hyperalgesia, characterized by lowered pain thresholds and heightened responses. Conversely, reduced sensitivity indicates a loss of sensory function, resulting in hypoesthesia or hypoalgesia, which corresponds to elevated nociceptor activation thresholds.

In humans, mechanical and thermal pain thresholds were validated for their reliability, specificity in distinguishing OA from non-OA individuals, and sensitivity including responsiveness to treatment (7, 10, 12, 61–63). Mechanical PWT was validated in experimental OA models in rats (9, 15, 16, 41–45) and in natural OA models in cats (64, 65) and dogs (66), as well as experimental OA (46) and cancer (67) in dogs. Mechanical PWT was assessed using an electronic von Frey Esthesiometer® (IITC Life Sciences Inc., Woodland Hills, CA, USA) with values recorded in grams. By measuring repetitively mechanical thresholds (test-retest to characterize the outcome repeatability), data distribution allowed to classify lower values, first quartile more exactly, as highly sensitized, corresponding to an allodynic status. It was established that OA cats with at least two values (on a minimum of four measurements) below the established first quartile threshold for either thoracic or pelvic limb values are considered allodynic (64). Thermal PWT was described in studies involving rats, cats and dogs, but led to more variability and less reliability than the mechanical threshold (8, 9, 11, 68). PPT was validated in rats using either a pressure application measurement device (69, 70) or the Randall-Selitto electronic algesimeter (IITC 2500 Digital Paw Pressure Meter, IITC Life Science, Woodland Hills, CA, USA) in grams (71). In dogs, PPT was validated using a pressure algometer (Wagner Force Gauge M3-2, Mark-10 Corporation, Copiague, NY, USA) with measurements recorded in Newtons (N) (11). For both mechanical PWT and PPT assessments, pressure is gradually increased until an aversive behavioral response is observed (paw withdrawal, agitation, weight shift, vocalization, licking, avoidance, etc.) or until the cut-off value is reached (see Supplementary Appendix S1 for further details).

#### 4.1.2 Tactile sensitization—brushing

Brushing assesses Aβ-fibers sensitivity, and the potential transmission of aberrant pain signal at the affected or remote site. Although the precise mechanisms remain unclear, mechanoreceptors could undergo sprouting, switch their phenotype to enhance synaptic transmission, and WDR neurons could contribute to relay non-nociceptive stimuli as excitatory post-synaptic potentials in maladaptive pain (72). Historically, brushing was employed to assess receptive field WDR neurons firing rate in anesthetized rats and cats (73–76). It was later validated for bedside assessments in humans using a foam brush (77) and demonstrated specificity in dogs, although only one study has investigated its use in osteosarcoma dogs (67). Healthy individuals tolerate the maximum of passages, whereas sensitized patients, exhibiting gain of function, could perceive pain before reaching the maximum (i.e., hypoesthesia or allodynia). Brushing was performed at the primary or secondary source of pain until the individual indicated pain, an aversive behavior was observed in dogs, or the cut-off value was reached (4 passages) (see Supplementary Appendix S1 for further details).

#### 4.1.3 Central sensitization—response to mechanical temporal summation

**Figure 3:**
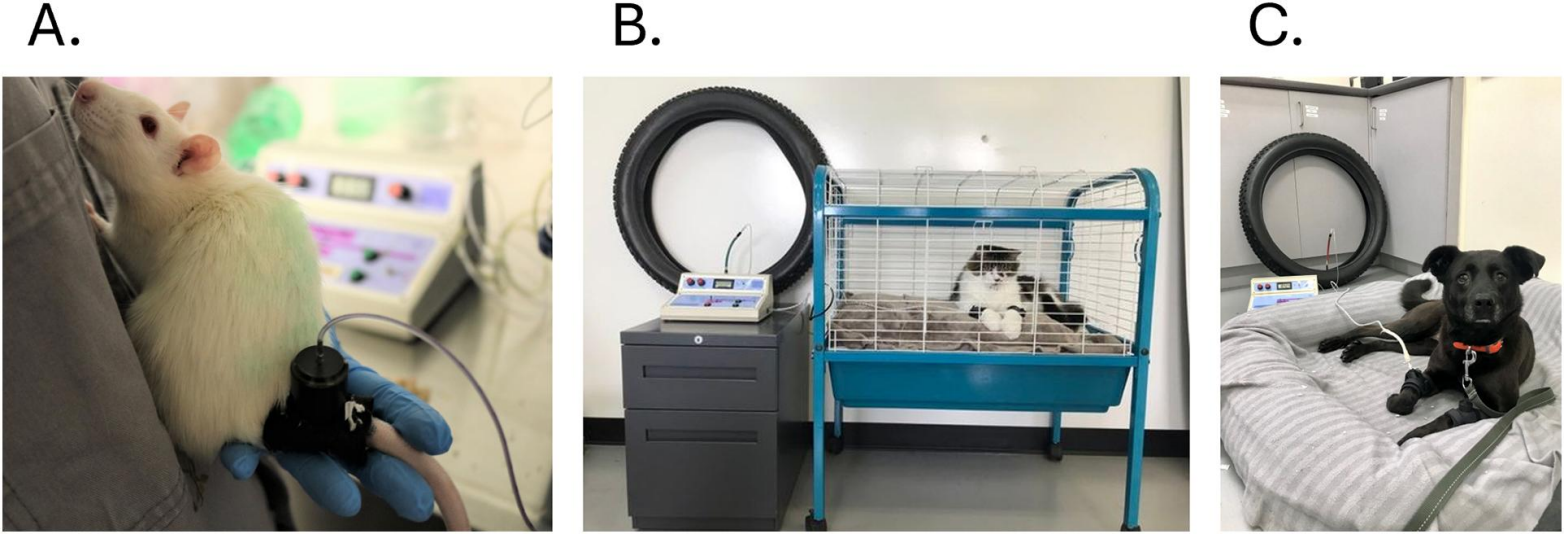
Photographs of the mechanical temporal summation set-up in rats **(A)**, cats **(B)** and dogs **(C)** the stimulation was placed at mid-forearm for cats and dogs but can also be placed at the tail-basis.

If wind-up is the initiating phase in spinal and central sensitization, consecutive temporal summation (TS) of noxious or non-noxious stimulus leads to enhanced neuronal excitability and increased pain perception, and can be evaluated (48, 49). The TS phenomenon occurs with a stimulus repetition at a frequency of 0.3–0.5 Hz to activate C-fibers. The cumulative AP generated leads to a series of amplifying mechanisms that ultimately manifest as primary hyperalgesia/allodynia, resulting from sensitization at the site of injury, and secondary hyperalgesia/allodynia, which reflects an expanded pain field (spatial summation) beyond the injured area (48). The release of substance P and glutamate enhances activation of α-amino-3-hydroxy-5-methyl-4-isoxazolepropionic acid (AMPA) and neurokinin-1 receptors on the post-synaptic neuron. The resulting rapid membrane depolarization removes the magnesium ion (Mg^2+^) blockade from *N*-methyl-D-aspartate (*N*MDA) receptors. The entry of glutamate via *N*MDA receptors boosts the summation of excitatory post-synaptic potentials (48). Calcium influx via ionophore postsynaptic *N*MDA receptors activates intracellular protein kinase C, increasing the probability of *N*MDA channel opening and reducing the voltage dependency of Mg^2+^ blockade (48). These mechanisms contribute to a long-lasting cumulative membrane depolarization and heightened neuronal excitability. During chronic pain, the wind-up phenomenon is not only mediated by C-fibers but is aggravated by Aβ-fibers. Due to neuronal plasticity and altered lamina innervation in the dorsal horn, non-noxious stimulus could therefore contribute to the pain experience and central sensitization (72). Historically, repeated electrical stimuli were used to mechanistically describe the wind-up phenomenon in anesthetized rats (49). While this approach elucidated the underlying TS mechanism, it did not capture the affective-motivational dimension of pain perception. In humans, TS was elicited by mechanical or thermal non-noxious stimuli and pain experienced recorded on a visual analogue scale or numeric pain scale after a predefined number of stimulations (10, 12, 61). The increase in nociceptive sensitivity induced by TS can last approximately from 15 s to 2 min for healthy individuals and may last longer with maladaptive pain (78–80). In conscious animals, the response to mechanical temporal summation (RMTS) was developed and validated first in OA cats (81) for its specificity (by distinguishing healthy and OA cats) (81, 82) and sensitivity (by its responsiveness to treatment) (82, 83). The RMTS methodology was subsequently adapted for rats (84), as well as for the canine natural OA model (85) (Figure 3). Repetitive mechanical stimuli were delivered using the Top Cat System (Top Cat Metrology®, Bespoke Measurement Systems, Cambridgeshire, UK) device, which enables standardized sub-nociceptive stimuli. Indeed, the intensity (in N), the frequency (in Hz) and the duration of the stimuli (in sec.) are fixed, according to the condition, with a cut-off value of 30 stimulations in OA animals (81). The number of stimulations delivered before an aversive behavior response (e.g., jumping, agitation, vocalization, biting or licking the device, avoidance) is recorded. It is expected that OA animals will tolerate a lower number of stimuli, reflecting a gain in sensory function with a sustained sensitivity even after the last stimulation (8, 16). Detailed explanations of the central sensitization assessment are provided in the Supplementary Materials (Supplementary Appendix S2).

### 4.2 Pain endogenous modulation

Central (dorsal horn, in particular) “gating” modulates which information is passed through the dorsal horn. It is self-modulation (inhibitory or facilitatory).

#### 4.2.1 Pain endogenous inhibition—conditioned pain modulation

**Figure 4:**
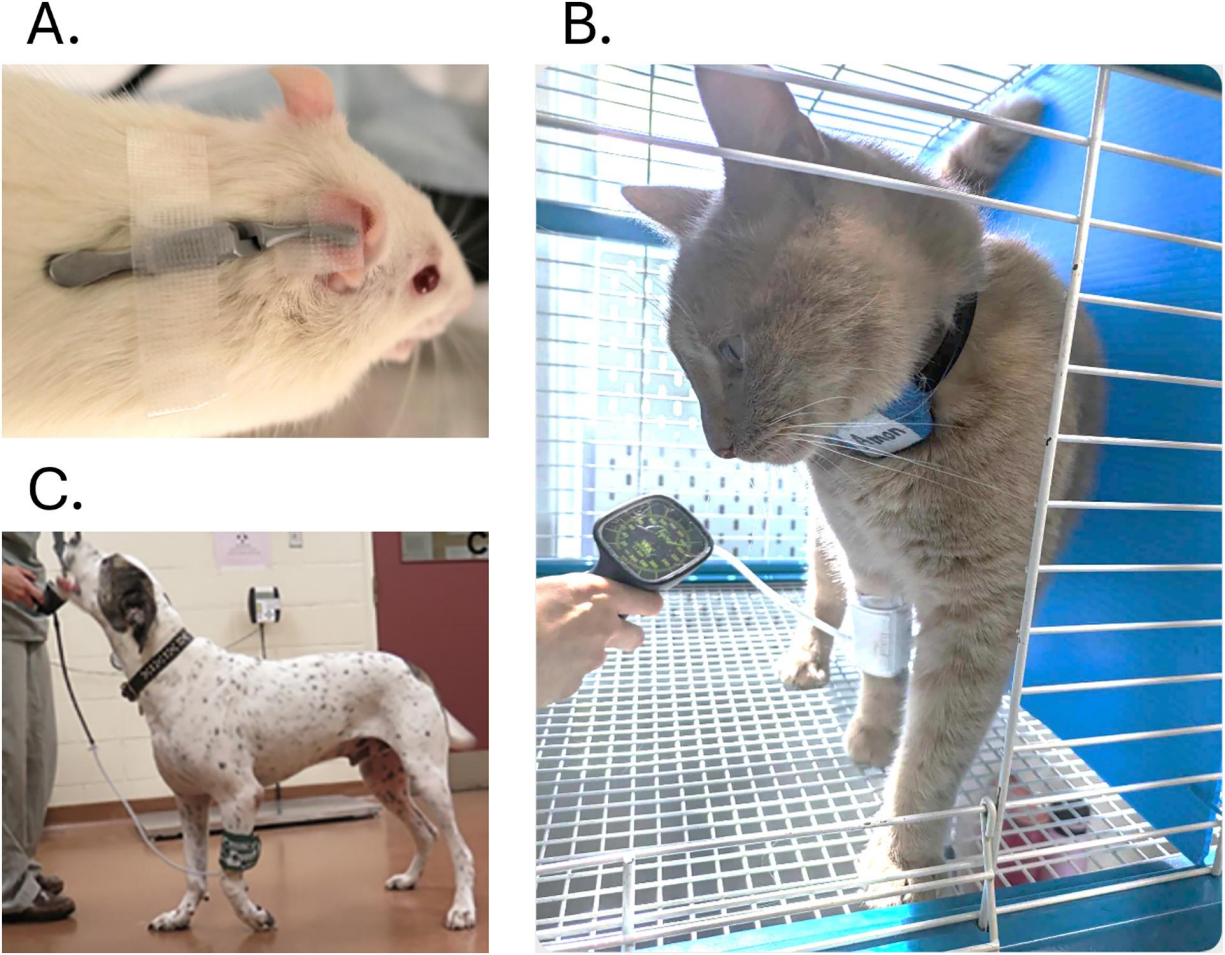
Photographs of the conditioned pain modulation set-up in rats **(A)**, cats **(B)** and dogs **(C)**

Endogenous descending pain inhibition in both humans and animals is a key component of physiological and pathological pain processing (see above chapters 2 and 3). Diffuse noxious inhibitory controls (DNIC) were measured as the inhibition of second-order WDR neurons following the application of a noxious stimulus outside the receptive field of the recorded neuron (86, 87). DNIC represents a fundamental pain-modulating system, initially identified in animal models and later reported in humans. It functions as a self-regulatory mechanism, whereby one pain stimulus inhibits another, commonly referred to as “pain inhibits pain”. Conditioned pain modulation (CPM) was subsequently introduced as a method to evaluate the DNIC system in conscious individuals (88). It refers to a reduction in the pain perception from a test stimulus when a noxious conditioning stimulus (CS) is applied to a body site remote from the primary pain (i.e., “pain inhibition by pain”). In humans and animals, the test stimulus can be PPT/PWT measured before (Pre_CPM) and during or immediately after the CS (Post_CPM). A mechanical, ischemic or thermal CS was validated in humans (88, 89), whereas mechanical or ischemic CS was validated in rats (16, 57, 84, 88, 90), cats (91) and dogs (67, 85, 92, 93) (Figure 4).

The inhibition rate was calculated based on Formula (1). A rate >0% indicates functional endogenous pain inhibition (i.e., reduction in pain following the CS application), and the animal was considered as a positive responder with the I/F balance in favor of inhibition. In humans a rate >±2x Standard error of measurement (SEM) based on healthy individuals was suggested to determine the responsiveness (94). Further details on CPM evaluation are provided in the Supplementary Files (Supplementary Appendix S3).

Formula (1)

$$\mathrm{Inhibition}\;\mathrm{or}\;\mathrm{facilitation}\;\mathrm{rate}\;(\text{\%})=\frac{\mathrm{Post}\_\mathrm{Measure}-\mathrm{Pre}\_\mathrm{Measure}}{\mathrm{Pre}\_\mathrm{Measure}}\times 100\;$$

One open question regarding the mechanisms of DNIC concerns the precise functional identities of WDR neurons (95). It is not clear whether these are inhibitory or excitatory, local or projection neurons (96), and they have not been assigned to any molecular classes of neurons defined using single-cell RNA sequencing (97). It is further unclear how inhibition of WDR neurons, found within lamina V of the dorsal horn, translates into changes in pain perception when the nociceptive-specific region of the spinal cord is considered to be within superficial laminae (98). Fundamentally, the exact mechanism of WDR inhibition is unknown and could involve direct postsynaptic inhibition, engagement of inhibitory spinal circuits, or presynaptic inhibition of the nociceptive afferent input onto WDR neurons (99). Efforts to further advance the understanding of DNIC mechanisms should be paralleled by mechanistic investigations of the human counterpart, CPM. One major challenge is the sheer abundance of CPM methods hindering comparability, meta-analytical approaches, and generalizability (11). Developing standardized protocols for multiple test stimuli/conditioning stimuli combinations would improve comparability between studies (99). Finally, CPM effects are mainly explained by interindividual differences other than age, sex, and conditioning stimulus intensity (100), and their origin (e.g., psychological or genetic) requires further investigation.

#### 4.2.2 Pain endogenous facilitation—temporal summation

Temporal summation of pain is a human proxy for wind-up of dorsal horn neurons as assessed in animals. The standard paradigm for eliciting TS of pain involves repetitive nociceptive stimuli of equal intensity (101). The wind-up phenomenon can be modulated by descending pain control mechanisms, with an imbalance occurring during chronic pain between I/F leading to pain facilitation (49). The latter is assessed by testing the second pain or neuro-sensitization that occurs following TS-induced wind-up. In humans, the pain experienced before and after wind-up induction was typically self-reported using a pain scale, with an increase indicating facilitation (10, 61). However, this reported facilitation was entirely subjective and did not necessarily reflect neuro-sensitization, as QST was not performed before and after wind-up induction. In animals, the use of pain scales is subjective, influenced by the evaluator and subject to the caregiver placebo effect, necessitating the use of other objective tools (3). In rats, only one article demonstrated that TS (with thermal stimuli) increased nociceptive sensitivity, illustrated by a shorter tail withdrawal latency post-TS (78). Additionally, a GREPAQ symposium presentation reported increased mechanical nociceptive sensitivity (decreased PWT) one minute after RMTS (84). Although no published articles have yet examined facilitation induced by mechanical TS in cats and dogs, the RMTS device (Figure 3) could be suitable for inducing TS (as a sustained neuro-sensitization was observed) and a PWT/PPT could be measured before and after stimulations (8, 11). Facilitation rate was calculated using Formula (1). A rate < 0% indicates pain endogenous facilitation (i.e., increased pain following the RMTS stimulation), and the animal was considered as a positive responder with the I/F balance in favor of facilitation. Further details on TS evaluation are provided in the Supplementary Files (Supplementary Appendix S4).

A recent scoping review of TS measurement in adults with musculoskeletal conditions and healthy individuals identified 250 studies using mechanical stimuli, and 125 using thermal stimuli (101). Across these studies, forty-six different instruments were employed. Repetitive stimuli were applied using 31 different frequencies (ranging from 0.03 to 200 Hz) and stimulation duration varied from 5 to 1090 s. As with CPM protocols, TS of pain methodologies exhibit substantial variability, complicating the comparison and synthesis of results.

## 5 Discussion

### 5.1 The osteoarthritis phenotypes revealed by quantitative sensory testing

#### 5.1.1 There are many osteoarthritis phenotypes in humans, but what about animals?

The investigation of OA pain mechanisms in humans has revealed heterogenous OA pain phenotypes, based on neuro-sensitization and I/F modulation evaluations (13, 14), which influence therapeutic efficacy (12). Indeed, neuro-sensitization was correlated with the experienced pain, positioning QST as a valuable tool for phenotype identification (102). A recent exploratory study described four distinct phenotypic profiles in patients with knee OA (*N* = 134), compared to healthy individuals, based on I/F balance (14). The pro-nociceptive pain profile characterized by increased TS and decreased CPM was the most prevalent (41%); In contrast, the anti-nociceptive pain profile, defined by decreased TS and increased CPM, was the least common (9%). Two intermediate profiles were also reported: increased TS and (responsive) CPM (16%), and decreased TS and (preserved) CPM (34%). Systematic reviews with meta-analysis have similarly identified the pro-nociceptive pain profile (decreased PPT, increased TS and decreased CPM) as the predominant phenotype among OA patients (7, 103, 104). Furthermore, decreased (fatigue) CPM correlated with increased OA pain intensity, as measured by the Western Ontario and McMaster Universities Osteoarthritis Index (WOMAC) (105). Recently, Saxer et al. published a review referencing 11 articles and reported multiple phenotypes in patients with knee OA (13). These profiles were determined by the level of neuro-sensitization, assessed by QST (8/11 articles) and by individual characteristics (comorbidities, anxiety, depression, sleep, activity), taking into consideration the multidimensional aspect of OA chronic pain (13). Although the classification of profiles varied across studies, the pain sensitivity clusters identified through QST were consistent with the four pain profiles described by Petersen et al. (14).

In animals, studies reporting a pain phenotypic profile characterized by (peripheral and central) sensitization and pain modulation (I/F balance) remain rare. Only one article, published by the GREPAQ, has described a sensitization profile in OA rats, laying the groundwork for future phenotyping in animals (16). The study demonstrated persistent peripheral sensitization (decreased PWT), transient central sensitization [decreased RMTS at Day (D) 21 only], and fatigue of pain endogenous inhibitory control (decreased CPM at D56) in the surgical OA rat model (16). In cats, a proposed profile was based on the first quartile of PWT to distinguish highly vs. minimally neuro-sensitized OA cats. Allodynic cats were significantly more sensitized compared to healthy and non-allodynic cats (64), later confirmed by Lefort-Holguin et al. (91). Further, according to a validated clinical metrological instrument [i.e., subjective MI-CAT(V) pain scale], moderately and severely affected OA cats exhibited significantly lower PWT values compared to healthy and mildly affected OA cats (91), similar to human studies (13). Most studies have focused on either neuro-sensitization or -modulation. Peripheral sensitization has been widely demonstrated in OA models, either (chemically or surgically) induced in rats (9, 15, 16, 41–45), or in dogs (46), and in naturally occurring OA in cats (8) and dogs (11) (decreased PWT/PPT vs. control groups and/or baseline value). Fewer studies have addressed the characterization of central sensitization in animals. Evidence of central sensitization in OA rats was limited: showing only transient TS (16) or enhanced tactile sensitization in anesthetized individuals (74–76). In contrast, central sensitization via mechanical TS was observed in OA cats meta-analysis (8) and in dogs with osteosarcoma (increased tactile sensitization) (67) but remained controversial in OA dogs meta-analysis due to lack of homogenous data (11). Central sensitization was associated to an up regulation in spinal pro- and anti-nociceptive neuropeptides, neuroepigenetic and pro-inflammatory markers, as well as an overactivity of glial cells in OA rats (15, 16, 41, 42, 44, 45, 106) and an increase in spinal substance P in experimental OA dogs (46).

Regarding the evaluation of pain endogenous modulation, some published studies seem promising for phenotypic pain profiling in animals. In rodents, the evolution of CPM was observed at different stages of MIA-induced OA pathology (57, 90). At early OA stage, rats exhibited functional CPM comparable to control animals, whereas CPM was abolished at a late stage (corresponding to chronic stage in humans). A recent study confirmed activation of pain endogenous inhibitory control (increased CPM) during the early phase (at D21 after surgical OA induction) which faded over time in the Montreal Induction of Rat Arthritis Testing (MI-RAT^©^) model, in correlation with increased neuronal activity and histological scores (16). Another study using the MI-RAT^©^ model demonstrated that CPM responses were modulated by aging. In healthy aging, represented by the LOU/C/Jall rat strain, endogenous inhibitory control was preserved at D7, D21, and D60 after OA induction, whereas in sedentary aging, represented by the Sprague-Dawley strain, CPM was progressively impaired (107). More precisely, the MI-RAT^©^ model demonstrated both peripheral (decrease in PWT by −20%) and central (decrease in RMTS count by −50%) sensitization, indicative of enhanced TS and corresponding responsive CPM. These features were observed in 90% of OA rats throughout most of the follow-up period, but CPM fatigued at timepoint D83 (being active in only 50% of OA rats) (75), reflecting a shift toward a pro-nociceptive profile characterized by reduced descending inhibition and increased pain facilitation over time. Importantly, as a preclinical and standardized model, the MI-RAT^©^ aims to investigate OA pain mechanisms under controlled laboratory conditions. Its use enables a comprehensive temporal characterization of OA pain pathophysiology, encompassing both peripheral and central sensitization processes as well as the evolution of the I/F balance. Furthermore, it serves as a translational step for validating QST metrological properties (e.g., concurrently to spinal neuropeptides) before their application in naturally occurring OA in dogs and cats.

In natural OA models in dogs and cats, a similar neuro-sensitization profile was observed, with −20% to −30% decrease in PWT/PPT and −35% to −75% decrease in RMTS count compared to healthy controls. This corresponded to enhanced TS and responsive CPM, present in 60% of OA animals (85, 91). In a longitudinal OA dog follow-up (2–3 years), the increase in neuro-sensitization was associated with functional CPM until the onset of fatigue and engaged prognosis in several OA dogs (85). These findings confirmed earlier preliminary evidence of CPM fatigue in a small sample of OA dogs compared to controls (93). These studies underscore the fatigue of endogenous inhibitory control in the OA chronic phase for animals, mirroring the natural progression of the pathology in humans. Unfortunately, few studies have evaluated pain endogenous inhibition in healthy and OA cats (8, 91). Similarly, preliminary studies have started investigating the pain endogenous facilitation in animals (84, 85, 91), whereas in humans, it has been more extensively assessed using pain scale rating before and after TS (10). However, based on recently published results, the pro-nociceptive pain profile (decreased PPT/ PWT, increased TS and decreased CPM) appears to be the predominant phenotype in OA rats (laboratory-induced model), as well as in OA cats and dogs (natural models), following an initial phase of responsive inhibitory control to neuro-sensitization.

**Figure 5:**
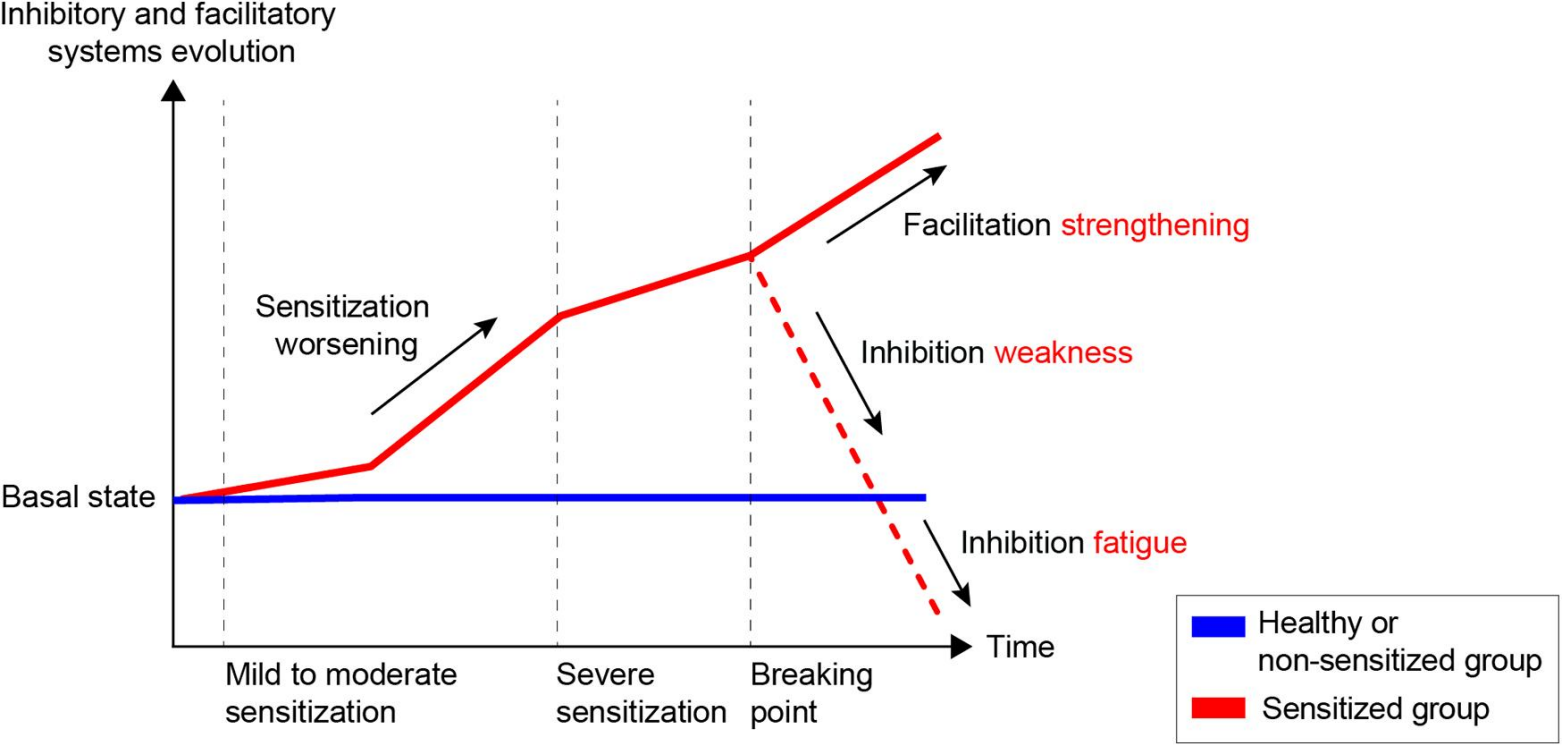
Schematic graphic of pain modulation balance between inhibitory and facilitatory systems in healthy or non-sensitized (blue) and sensitized (red) patients.

A translational hypothesis can be formulated regarding the imbalance between I/F mechanisms in chronic OA pain across humans and animals, as illustrated in Figure 5. In the early stages of the disease, OA patients activate their endogenous inhibitory controls (increased, responsive CPM) to counterbalance the facilitation (decreased PPT/PWT and enhanced TS) allowing them to maintain comfort in daily activities (13, 14, 16). As chronic pain develops, severely affected patients gradually lose the effectiveness of inhibitory control (decreased CPM), reaching a breaking point where the system fatigues and facilitatory mechanisms dominate. This hypothesis was demonstrated in OA humans and partially in OA rats, but it still needs to be validated in OA cats and dogs.

#### 5.1.2 Improving quantitative sensory testing standardization

As described in Table 2, the majority of QST outcomes have been validated in rats (for mechanistic understanding of OA pain in basic research) and in humans (to better understand the pain experience). Although studies in cats and dogs remain limited, published results suggest promising inter-species translation of available QST protocols. To improve the reliability of these tests, increasing the number of trials per measure has been proposed. For example, in healthy subjects, duplicate PPT and triplicate TS measurements reduced error and demonstrated excellent relative reliability compared to single-trial measurements (108). Although static QST such as pain thresholds (PWT, PPT, etc.) are reliable, evidence for the reliability of so-called dynamic QST, particularly for CPM, remains inconclusive (89, 109, 110). It is worth noting that a systematic review (80) included only four studies, each assessing different types of chronic pain and using varied CPM stimuli, which contributed to high variability in results. To investigate pain phenotype, most studies evaluated the I/F balance via the assessment of CPM (inhibition) and TS (facilitation) (7, 14, 103, 104). However, the correlation between these two assessments has not yet been explored, and some studies have even shown no correlation between TS and CPM in healthy subjects (111). This suggests distinct brain mechanisms for I/F and that their relationship may be more complex than a linear regression. A recent systematic review indicated that insufficient methodological standardization (only 9% used referenced guidelines) and data heterogeneity (e.g., in relation with different location of assessment, small population of individuals, methodological biases, etc.) precluded the conduct of a meta-analysis, resulting in limited insight into the degree of sensitization in OA dogs (11). In OA cats, current studies report rather reliable and sensitive results, which seem promising even if some aspects, particularly CPM, are missing (Table 2) (8). Age and sex are important risk factors to consider in the assessment of chronic OA pain (1, 112). The OA prevalence increases with age and is more common in females, with elderly female being more affected by this pathology (2, 112). Some studies have demonstrated the impact of these factors on QST results in rodent pain models (113, 114). Moreover, a meta-analysis showed that aging disrupts the I/F balance, in favor of facilitation (pro-nociceptive profile with increased TS and decreased CPM) in healthy individuals (109, 115). It is important to explore the evolution of the painful OA phenotype with aging. Moreover, it is essential to improve the standardization of assessments of the painful OA phenotype by QST to allow an objective, reliable, repeatable and reproducible measurement in animals, mimicking the human condition (17). This standardization must include rigorous protocols for acclimatizing patients to testing procedures and environmental conditions to minimize stress-induced analgesia or hyperalgesia, well described in the literature (for more information, see the Supplementary Appendices S1–S4) (116).

### 5.2 Improving osteoarthritis pain management through quantitative sensory testing sensitivity

The management of OA pain in both human and veterinary medicine encompasses non-pharmaceutical approaches, pharmaceutical therapies, and surgical interventions. According to a 2020 review of guidelines from the European Society for Clinical and Economic Aspects of Osteoporosis, Osteoarthritis, and Musculoskeletal Diseases (ESCEO) and the Osteoarthritis Research Society International (OARSI), core treatments include education, structured exercise and weight loss, with pharmacological interventions and, in severe cases, surgery is recommended for pain management (117). Canadian consensus guidelines for canine OA follow a similar approach based on the Canine OsteoArthritis Staging Tool (COAST) (118). In cats, a 2023 review on feline OA management advocates for an integrative, multimodal strategy, incorporating anti-inflammatory and analgesic medications, dietary modifications, nutraceuticals, environmental adaptations, and physical rehabilitation (119). However, many recommendations for feline OA are extrapolated from other species, lacking direct scientific evidence (3).

Although systematic studies on neuro-sensitization in OA pain date back approximately ten years for humans and rats (120) and about five years for dogs and cats (8, 11), the effectiveness of various treatments in addressing OA-associated neuro-sensitization remains unclear. Non-pharmacological therapeutic approaches were not detailed in this section, given the current lack of QST-based evidence assessing their effects on neuro-sensitization in cats and dogs. This section of the review aims to summarize how standard OA pharmacological treatments may influence neuro-sensitization modulation (Table 3). Significant therapeutic effects on neuro-sensitization have been documented in both humans and animals with various pharmaceutical interventions, including non-steroidal anti-inflammatory drugs (NSAIDs), opioids, gabapentinoids, corticosteroids, anti-NGF monoclonal antibodies (mAbs) and other anecdotal treatments. NSAIDs are currently the first-line treatment for managing OA pain in humans (117), dogs (118), and cats (121). In human studies, etoricoxib (62, 122) and a combination of ibuprofen and acetaminophen, with or without pantoprazole (123, 124), have been investigated, suggesting a potential reduction in peripheral and spinal sensitization. Similarly, in rat OA models, NSAID treatment (e.g., carprofen) has been associated with decreased neuro-sensitization, as evidenced by improvement in PWT value, and downregulation of pro-nociceptive neuropeptides (43). The effects of NSAIDs on feline OA neuro-sensitization have been evaluated in two studies. Guillot et al. assessed the efficacy of different meloxicam dosages over four weeks in cats with naturally occurring OA and no changes in PWT threshold were detected, when the meloxicam group with lower responsiveness to treatment presented the higher percentage of allodynic cats (64). Likewise, Monteiro et al. reported no meloxicam effect on spinal sensitization, as reflected by unchanged RMTS counts during treatment (83). Guillot et al. observed that the efficacy of meloxicam might depend on the distribution of neuro-sensitized cats within treatment groups, indicating that allodynic cats may still experience persistent pain despite NSAID administration, whereas non-allodynic cats responded better, showing increased locomotor activity and improved limb function (64). In a recent study (GREPAQ—Internal data), allodynic cats were more represented in moderate and severe OA clusters, while non-allodynic cats were in the mild OA cluster. Moreover, firocoxib showed beneficial effects on neuro-sensitization and functional alterations, particularly in non-allodynic OA cats, who were more susceptible to a negative rebound post-firocoxib withdrawal. These findings underscore the importance of clustering patients based on neuro-sensitization status to optimize pain management across different phenotypes. Nevertheless, some NSAIDs are believed to exert central effects by either blocking the arachidonic cascade directly at the dorsal horn (for those able to cross the blood-brain barrier) or by activating pain descending inhibitory pathways (125, 126). However, gastrointestinal, cardiovascular, hepatic or renal adverse effects were described in human and animal patients (117, 121, 127).

**Table 3:** QST sensitivity to OA pain treatments. A positive effect (i.e., a decrease in sensitization) is reflected by either an increase (↑) in PPT or PWT or (↓) thermal hyperalgesia (TH), a decrease (↓) in TS and/or an increase (↑) in CPM. Ø, No effect on the outcome; PO, Oral administration; N/A, Not available; SC, Subcutaneous injection; IP, Intraperitoneal injection; SI, Systemic injection; SPI, Spinal injection; IA, Intra-articular injection.

Species	Results	Intervention (dose if applicable)	Reference
Non-steroidal anti-inflammatory drugs (NSAIDs)			
Inhibition of the cyclooxygenase (COX) enzymes and subsequent production of eicosanoids			
Rat	↑ PWT	Celecoxib (30 mg/kg/day) PO	(120, 190)
	Ø PWT, Ø CPM	Celecoxib (3 mg/kg/day) PO	(90)
	Ø PWT, Ø PPT, Ø TH	Rofecoxib (10 mg/kg) PO	(120)
	↓ TH, Ø PWT	Meloxicam (1–10 mg/kg/day) PO or IP	(191, 192)
	↑ PWT, ↑ PPT	Naproxen (10 mg/kg/day) PO	(70)
	↑ PWT	Loxoprofen (2.8 mg/kg) or Ketoprofen (1.1 mg/kg) patch	(193)
	↑ PWT	Carprofen (5 mg/kg/day) SC	(43)
	↑ PWT	Aspirin (75 mg/kg) PO	(190)
	↑ PPT, Ø PWT	Diclofenac (30 mg/kg) SC	(120)
Cat	Ø PWT, Ø TS	Meloxicam (0.025–0.05 mg/kg) PO/day	(64, 83)
	↑ PWT, ↓ TS	Firocoxib (0.15–0.4 mg/kg) PO/day	(GREPAQ internal data)
Dog	N/A		
Human	↑ PPT, ↓ TS, Ø CPM, ↑ PcPT	Etoricoxib (60 mg/day) PO	(62, 122)
	Ø CPM	Ibuprofen (1.2 g/day) + Acetaminophen (3.0 g/day) PO	(124)
	↓ TS for responders	Ibuprofen (1.2 g/day) + Acetaminophen (3.0 g/day) + Pantoprazole (20 mg/day) PO	(123)
Corticosteroids			
Inhibition of COX-2 action and phospholipase-A2 by inducing lipocortins			
Rat	↑ PPT	Triamcinolone acetonide (40 mg/mL) IA	(194)
Cat	N/A		
Dog	N/A		
Human	↑ PPT or Ø PPT, Ø TS	Lidocaine (10 mg) + Methylprednisolone (40 mg) IA	(138, 139)
Anti-nerve growth factor (NGF) monoclonal antibodies			
Neutralization of NGF activity and NGF/TrkA signaling			
Rat	↑ PWT	Inhibitor of TrkA kinase activity (30 mg/kg/2xday)	(140)
	Ø or ↑ PWT	Anti-NGF mAb (1–10^4^ μg/kg/week)	(140, 141)
	Adverse effects: skin irritation		
Cat	↑ PWT, ↓ TS	Frunevetmab (3 mg/kg) SC	(GREPAQ, internal data)
Dog	↑ PWT	Bedinvetmab (1 mg/kg) SC	(GREPAQ, internal data)
Human	N/A		
Opioids			
• Agonists at the μ-opioid receptors resulting in inhibition of neurotransmission			
Rat	↑ PWT, ↑ PPT, ↓ TH	Morphine (2–6 mg/kg) SC	(43, 120, 192)
	↑ PWT, ↓ TH	Morphine + Oxycodone (0.75–3 mg/kg) IP	(195)
	Ø or ↑ PWT, ↑ CPM	Tapentadol (1–5 mg/kg) SC	(57)
	↑ PWT, ↑ CPM	Tapentadol (1 or 2 mg/kg) + Pregabalin (5 or 10 mg/kg) SC	(196)
	↑ PWT	Tramadol (10 mg/kg/day) IP	(133)
Cat	↑ RMTS	Tramadol (3 mg/kg) PO/12h	(65, 82, 83)
Dog	N/A		
Human	↓ PPT	Opioid (without more information)	(129)
	↑ PPT, Ø TS, Ø CPM	Hydromorphone (4 mg) PO	(146)
	Ø TS	Tapentadol (50–250 mg/day) PO	(197)
Gabapentinoids			
Inhibition of the high-voltage-activated calcium channel			
Rat	↑ PWT, ↓ TH	Gabapentin (10–100 mg/kg) PO/2xday or SC or IP	(120, 192)
	↑ PWT, ↓ TH, ↑ Brush, Ø CPM	Pregabalin (0.3–30 mg/kg) PO/day or SC or SI	(43, 57, 90)
	↑ PWT, ↓ TH, ↑ Brush, ↑ CPM	Pregabalin (5 or 10 mg/kg) + Tapentadol (1 or 2 mg/kg) SC	(196)
	↑ PWT, ↓ TH, ↑ Brush	Pregabalin (10 mg/kg) SI + Ondansetron, 5HT_3_ antagonist (10–50 μg/50 μL) SPI that blocked the descending serotoninergic facilitation	(74)
Cat	↑ PWT	Gabapentin (10 mg/kg) PO/8h	(65)
Dog	N/A		
Human	Ø PPT	Pregabalin (150 mg/day) PO	(136)
Acetaminophen			
Exact mechanism unknown Possibilities: weak inhibitor of peripheral COX and indirect activation of endocannabinoid system (via CBR1) in the CNS			
Rat	Ø PWT	Paracetamol (300 mg/kg) PO	(120)
Cat	N/A		
Dog	N/A		
Human	Ø CPM	Ibuprofen (1.2 g/day) + Acetaminophen (3 g/day) PO	(124)
	↓ TS for responders	Ibuprofen (1.2 g/day) + Acetaminophen (3 g/day) + Pantoprazole (20 mg/day) PO	(123)
Selective serotonin/norepinephrine reuptake inhibitors and tricyclic antidepressants			
Block serotonin and norepinephrine reuptake transporter in a concentration-dependent manner			
Rat	↑ PWT, ↑ CPM	Duloxetine (10 or 20 mg/kg) SC or IP	(90, 198)
	↑ PWT	Duloxetine and Vortioxetine (2 mL/kg/day) PO	(199)
	↑ PWT	Milnacipran (10 mg/kg) IP	(200)
	Ø PWT	Amitriptyline (3–30 mg/kg) IP	(192)
Cat	N/A		
Dog	N/A		
Human	Ø PPT, Ø TS, Ø CPM	Duloxetine (30–60 mg/day) PO	(136, 137)
Cannabinoids			
Activation of endocannabinoid receptors (CB1 and CB2)			
Rat	↑ PWT, ↑ PPT/PAM	CB2 agonist (1 and 5 mg/kg/day) IP or SC	(143, 145)
	↑ PWT	Cannabidiol (100–300 μg/100 mL) IA	(144)
Cat	N/A		
Dog	N/A		
Human	Ø PPT, Ø TS, Ø CPM	Dronabinol (10 mg) PO	(29, 146)
Arthroplasty			
Surgical joint replacement			
Rat	N/A		
Cat	N/A		
Dog	↑ PWT	12 months after surgery	(153)
Human	↑ PPT, ↑CPM, ↓ TS	3 to 18 months after surgery	(55, 151, 152, 201)

A meta-analysis by Suokas et al. on analgesic efficacy in OA rodent models found that opioids provide greater pain relief than NSAIDs, as evidenced by improvements in PWT assessments and reductions in spontaneous pain (120). However, opioids are generally not recommended due to their limited long-term efficacy and the risk of adverse effects, including opioid-induced hyperalgesia, which has been documented in both rats and humans (128, 129). The role of pain sensitization in the development of opioid-induced hyperalgesia in knee OA remains unclear (129). The management of this adverse effect can be possible with administration of *N*MDA receptor antagonist (e.g., ketamine) or antidepressants (e.g., amitriptyline) (130). Yet approximately 10%–20% of human patients continue to use opioids for OA pain management, but opioid-induced hyperalgesia modulation has not been specifically studied in this population (131).

Tramadol and tapentadol are considered atypical opioids as they function mainly as μ-opioid receptor agonists and norepinephrine reuptake inhibitors (132). This dual mode of action allows for limited incidence of opioid-induced side effects and pro-analgesic effects on central sensitization through the noradrenergic pathway (132). Both drugs have been found to improve allodynia/hyperalgesia in OA rat models (57, 133) and tramadol has been shown to effectively reduce TS in cats, suggesting its potential to reverse spinal sensitization and improve biomechanics in OA-affected individuals (65, 82, 83). Tramadol has not been tested in OA dogs for neuro-sensitization, but in this species the main active metabolite of tramadol is N-desmethyltramadol (M2), a norepinephrine reuptake inhibitor. Therefore, M2 will reinforce the pain descending inhibitory controls, but for this a minimum of three weeks of administration is required, and in such conditions, its association to NSAID was shown to be synergic (134).

Additionally, the release of pro-nociceptive neuropeptides has been shown to respond to various analgesics, with systemic pregabalin and morphine producing correlated changes in pain phenotype outcomes (43). The therapeutic effects of analgesics on CPM have been sparsely studied, focusing on opioids (morphine and tapentadol) and gabapentinoids (pregabalin and gabapentin) in OA rat models (57, 90). While opioids have been found to activate CPM in rats, the effects of gabapentinoids remain inconclusive. However, gabapentin (10 mg/kg PO q8 h over 30 days) has been shown to slightly improve peripheral sensitization and locomotor activity levels in a small sample of OA cats (65), when no analgesia and sedation were observed with a reduced dosage (10 mg/kg PO q12 h over 14 days) (135). No study on effect of gabapentinoids in OA dogs has been published, yet.

In the management of complex or refractory pain conditions, serotonin/norepinephrine reuptake inhibitors may be prescribed for their dual analgesic and antidepressant actions, as depression is a common comorbidity of OA-related pain (29, 117). While these drugs have been shown to reduce neuro-sensitization in rat models, they have not demonstrated significant effects in humans (136, 137), and data on their use in OA cats and dogs are lacking (Table 3). This highlights the need for further research to better understand their cross-species efficacy in OA management.

While corticosteroids effectively reduce inflammation by inhibiting cyclooxygenase-2 and phospholipase A2, their impact on OA sensitization mechanisms remains unclear as only the anti-hypersensitivity effect of intra-articular methylprednisolone with lidocaine has been tested in humans, with inconsistent results in PPT across studies (138, 139). New avenues are being explored with anti-NGF mAbs for pain management. In humans, these therapies have shown promising results in reducing pain; however, no studies specifically address their effects on neuro-sensitization (29). In animals, research on neuro-sensitization has been conducted exclusively in rat models (140, 141), highlighting again a gap in studies across species (Table 3). Interestingly, injection of frunevetmab in OA cats induced a significant increase in both PWT and RMTS (GREPAQ internal data) and a similar anti-neuro-sensitization effect was observed with bedinvetmab in OA dogs (GREPAQ internal data). However, due to the ubiquitous distribution and role of NGF, caution must be applied for detecting potential consequences of anti-NGF mAbs (long-term) administration. According to pharmacovigilance data, adverse effects appear to be rare (1–10 cases per 10,000 doses administered) (142). Studies aimed at identifying their risk in specific subpopulations, at describing the most adapted regimen of administration in OA (timing during the disease development, duration, association with other treatments, etc.) will be welcome.

Cannabinoid and nutritional-based interventions such as enriched therapeutic diets and nutraceutical supplementation remain poorly described in relation to their effects on neuro-sensitization (Table 3). So far, only four studies have investigated the efficacy of cannabinoids in OA management using QST. While these studies demonstrated an effect in OA rat models (143–145), no significant effects were observed in humans (29, 146). Furthermore, no studies have assessed the effects of cannabinoids on neuro-sensitization in cats and dogs, although existing research suggests a potential reduction in pain (118, 147). The potential efficacy of omega-3 fatty acids in modulating neuro-sensitization has been suggested due to the anti-inflammatory and neuroprotective properties, mediated by resolvins and protectins derived from eicosapentaenoic acid, docosapentaenoic acid and docosahexaenoic acid (3, 127, 147–149) and has been recently observed in OA cats with an enriched therapeutic diet (GREPAQ internal data).

In addition, physiotherapeutic modalities have been proposed for OA management, including (electro) acupuncture, laser therapy, extracorporeal shockwave treatment, and transcutaneous electrical nerve stimulation. However, to date, no studies have applied QST to evaluate their effects in cats and dogs (3, 127, 150). This highlights a current evidence gap and the need for objective QST-based approaches in blinded, prospective, randomized trials to better evaluate the effects of non-pharmacological therapies on chronic OA pain mechanisms across rats, cats and dogs. In the case of arthroplasty, several studies were published in humans, with a decrease in neuro-sensitization and improvement of pain modulation following total knee/hip joint replacement (55, 151, 152). One study in dogs reported an increased PWT threshold after total hip replacement in both operated and non-operated limbs after 12 months, indicating reduced central sensitization (153). No studies were reported in OA cats (Table 3).

A few studies involve therapeutics and QST (Table 3). Most used QST as a predictor of treatment response and were not directly concerned with the relief of neuro-sensitization. A pro-nociceptive profile (high TS and low CPM) was predictive of pain even after treatment or surgery (12, 55, 154). Around 20%–30% of the patients that have undertaken a total hip or knee arthroplasty reported pain after 3 months to 5 years (155). In dogs, the CPM functionality was predictive of NSAID or anti-NGF mAb treatment responsiveness in OA pain (85), whereas there are currently no studies in cats. Some limits should be raised from treatment studies, a control group was not always included [e.g. (153),], in rats most of time the analgesic follow-up was short [20 min to 24 h; e.g. (57, 120),] and, as few studies have been done, the majority come from the same research groups (in particular for humans, cats and dogs). It would be important to diversify the populations and treatments studied. Knowing the response of each neuro-sensitization cluster to several treatments (targeting biomechanical pain or sensitization) is of major importance in contributing to personalized medicine.

### 5.3 Complementary tests for a global understanding of the disease

#### 5.3.1 The importance of considering affective-motivational and cognitive dimensions

Since pain perception is a subjective and multidimensional experience, it is essential to establish a link between its sensory-discriminative and affective-motivational components and cognitive assessment in order to better understand its complexity (13). Changes in cortico-limbic structures (such as prefrontal cortex, hippocampus and amygdala) implicated in affective-motivational dimension and cognition were reported in human affected by chronic pain (156). The OA condition was associated with an increased risk of cognitive decline and dementia (157). Indeed, pain was predicted not only by QST (altered CPM and TS) but also by emotional and cognitive impairments (such as depression, anxiety and pain catastrophizing) in humans (13, 158). Translationally, late-stage MIA rats presented dysfunctional CPM accompanied by anxio-depressive state (159), and dogs with musculoskeletal disease presented more signs of cognitive impairments (160). As indicated in Table 3, the use of serotonin/ norepinephrine reuptake inhibitors could be of particular interest to decrease depression and indirectly alleviating experienced OA pain (159, 161). Greater optimism was associated with decreased TS, and decreased pain catastrophizing resulting in less severe clinical knee OA pain (162, 163). Separately, enhanced CPM was associated with greater optimism in healthy young adults (164) or significantly mediated the relationship between higher optimism and lower clinical pain severity (163). However, greater psychological resilience was associated with enhanced CPM in individuals with low optimism (i.e., pessimists) only (163). Optimism has also been assessed in dogs: individuals living in enriched environment or receiving positive human interaction showed more optimistic judgement biases, whereas dogs exposed to chronic stress tended to display pessimistic biases (165). Similarly, dogs experiencing neurological pain demonstrated more negative responses to ambiguous stimuli (166). Although optimism has not yet been directly linked to neuro-sensitization or descending inhibitory control in cats or dogs, it represents a promising avenue for improving our understanding of CPM functionality and the emotional component of pain.

This suggests the CPM reinforces the condition of painful persons only when its functionality is preserved. Future research should explore the effect of therapeutics (including dispositional optimism) on reducing TS (pain facilitation) and preserving CPM (pain inhibition), rather than merely activating, to maintain its functionality for the long-term well-being of patients. Efforts were made to find other methods of assessing central sensitization that are easier to implement clinically (e.g., sensory bedside testing, central sensitivity inventory scale) but further validation is required to replace QST (77, 167).

#### 5.3.2 From transmission to integration and perception of painful stimulation

By assessing pain perception, QST indirectly evaluates the conduction and integration of pain signal in the brain, in a semi-subjective way requiring the patient's cooperation (its reliability has been discussed above). A more direct evaluation could correspond to neuroimaging and electrodiagnostic tests. As mentioned in maladaptive pain chapter 3, results from neuro-imaging studies are conflictual with few evidence toward changes in brain structure or function between OA and healthy humans (51, 52). Neuro-imaging studies were rare in animals (53, 54) and no fMRI studies have been conducted to demonstrate neuro-sensitization or F/I imbalance.

Electrodiagnostic tests include nerve conduction studies (to assess the nerve integrity), electroencephalography (EEG; to assess cortical integration) and nociceptive withdrawal reflex (NWR; to determine spinal excitability). In human OA, only one article on nerve conduction was published and reported a decrease in sensory nerve AP amplitude, suggesting axonopathy and loss of nerve fibers functionality (168). Although this test is feasible in rats (169), cats (170) and dogs (171), to the best of authors knowledge, only one study was published with OA individuals. Declawed OA cats exhibited a decrease in hindlimb compound muscle AP amplitude compared to non-declawed OA cats (172); However, no comparison was made with healthy cats. The lack of studies could be explained by ethics and technological expertise concern, with the necessity of supramaximal electrical stimulation, perceived as painful, and performed under standardized anesthesia for animals. Even in neuropathic conditions, weak correlations were reported between QST and nerve conduction in humans (173). The complementarity of these two tests could be useful to better understand the gain/loss of sensory function occurring during OA, i.e., nerve conduction assesses large and myelinated fibers while QST assesses both large and small (un) myelinated fibers. The loss of sensory function may be indicative of demyelination, axonal degeneration, and/or intervention of pain modulatory I/F controls, whereas gain of sensory function may reflect neuronal hyperexcitability with intervention of pain modulatory I/F controls.

At the cortical level, evoked potentials (EP) recorded by EEG were synchronized with six types of QST stimulations assessing either Aβ-, Aδ- or C-fibers (174). The EP amplitude was sensitive and positively correlated with pain intensity perceived during mechanical or electrical stimulations in healthy individuals (175, 176). In OA cats, EP were synchronized with RMTS, but the small sample size prevented correlation analysis between amplitude and central sensitization (177). No comparative studies of sensory EP between healthy and OA humans, rats, cats or dogs were currently available in the scientific literature. However, a specific pattern of brain band oscillations was characterized for OA human patients and correlated with the pain experienced (178). Aberrant frequencies EEG oscillations were demonstrated at sensory discriminative, motivational-affective and descending inhibitory cortical regions in OA people (179). Notably, CPM effectiveness (i.e., reflecting descending inhibitory pathways) was negatively correlated with OA pain severity and relative power of delta brain oscillations (generated in pain control regions such as PAG) (105). These oscillations (such as recorded during CPM) were also predictive of post-operative opioid analgesia with 65% accuracy following hip joint replacement (180). However, in a neuropathic rat model, theta power and PWT predicted nociceptive states and were sensitive to analgesic administration of pregabalin, but without correlation between the two measurements (181). Further, spectral entropy, a non-linear feature of EEG analysis, was more effective at distinguishing neuropathic pain severity than linear features (i.e., band powers) in humans (182). This suggests its relevance in research on EEG neuro-sensitization biomarkers. Even if non-invasive EEG was feasible in OA rats (183), OA cats (177) and dogs (184) no studies assessed the brain oscillations pattern in response to sensitization in OA.

The third electrodiagnostic test, NWR, could be useful to correlate with QST. It assessed spinal hyperexcitability by applying repeated nociceptive stimulation, activate C- and Aδ-fibers and provoked an involuntary reflex (92, 185, 186). Facilitated TS of NWR was described in OA humans (185), OA rats (186) and OA dogs (92) compared to healthy individuals. It would be particularly interesting to correlate this reflex facilitation with TS, in conscious individuals, to distinguish the sensory-discriminative component of pain from others. Electrodiagnostic tests to characterize OA pain are in their infancy. Coupling them with QST looks promising for providing a complete picture of peripheral and central sensitization and/or altered pain modulation systems, and distinguishing phenotypes that may or may not be predictive of disease progression and management.

## 6 Conclusion

Neuro-sensitization has been studied in a translational manner across humans, OA-induced rat models, and in cats and dogs with naturally occurring OA using QST. A pro-nociceptive profile—characterized by increased neuro-sensitization, TS and decreased (fatigue) CPM—has been described in humans and suggested in rats, cats, and dogs, although further research is needed. The I/F balance appears to be a key factor in chronic OA pain, yet its progression over time remains to be characterized. Identifying pain phenotypes is particularly valuable for predicting treatment efficacy. While neuro-sensitization has been observed across species, there is a notable lack of studies evaluating the impact of analgesics on the I/F balance. Further research is essential to better characterize somatosensory changes in OA pain and develop personalized pain management strategies.
