# Supplementary material for: Translational validity of quantitative sensory testing in chronic pain neuro-sensitization: guide of use and interpretation in osteoarthritis animal models
Source: Front Pain Res (Lausanne). 2025 Dec 10;6:1709275. doi: 10.3389/fpain.2025.1709275 (PMC12728057; doi:10.3389/fpain.2025.1709275)
Supplement: Supplementary file 1 [file Datasheet1.pdf]

Appendix 1 – Peripheral sensitization

Université de Montréal

GREPAQ

Paw withdrawal threshold (PWT), pressure pain threshold (PPT) and tactile sensitization (brushing) (§ 4.1.1 & § 4.1.2)

Principle/Aim

To evaluate:

- Primary and secondary hyperalgesia and allodynia.
- Responsiveness of Aβ-fibers (PWT and brushing) or Aδ- and C-fibers (PPT).

Equipment

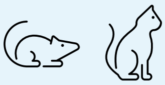

- Meshed cage
- Electronic von Frey® esthesiometer (max. 200g)

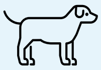

- Cushion/Comfortable bed
- Wagner® algometer (max. 10N)
- Soft brush (max. 4 passages)

Acclimatization (< 15 mins. per session)

Days	1	4	7	10	14
	Cage/contention Look at/sniff the device	Gentle touch with filament (PWT) or the hand (PPT)	Apply the device **without** pressure	Apply the device **with light** pressure	Apply the device **with normal** pressure

Step by step process

Basal/relaxed state →
- Place animal in the cage/on the cushion
- Give positive reinforcement (treats, affection)
- Wait few mins.

Device use * →
- Perpendicular application (paw, pad or metatarsus)
- Gradual increase or gently brush

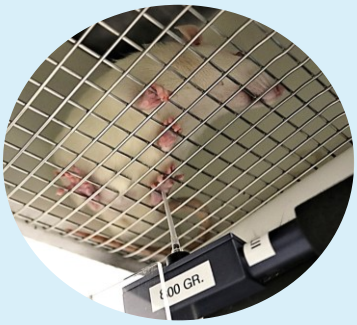

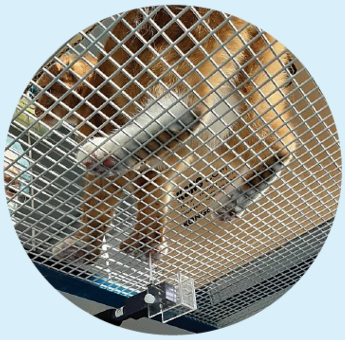

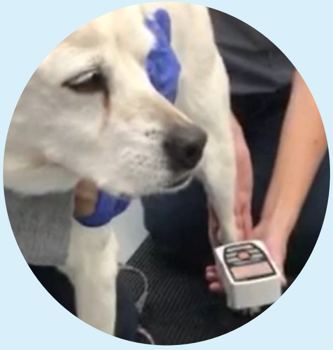

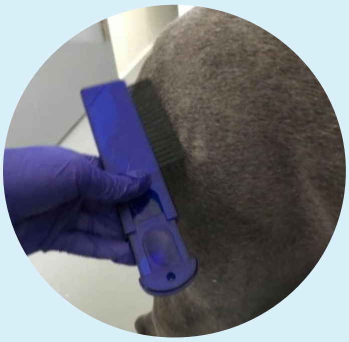

Take measure * →
- Observe aversive response (paw withdrawal, vocalization, *etc.*)
- Stop the device application
- Note the value

End of the experiment
- Give positive reinforcement
- Remove the animal
- Clean the cage or cushion

Interpretation

↓ PWT, PPT, brushing passages

Animal **with** peripheral sensitization

* Be careful, read before any assessment ⚠

- The animal MUST stand on its 4 limbs.
- If a reflex movement occurs when the device touches the skin this is not a valid threshold, take another measure once the animal has returned to its basal state.
- If necessary (agitation, stress) give some treats or petting to allow a return to basal state.
- No environmental distraction during the measure (noise, treat, light, *etc.*).

Electronic von Frey esthesiometer® from IITC Life Sciences Inc., Woodland Hills, CA, USA; Wagner Force Gauge M3-2 from Mark-10 Corporation, Copiague, NY, USA
