# Supplementary material for: Translational validity of quantitative sensory testing in chronic pain neuro-sensitization: guide of use and interpretation in osteoarthritis animal models
Source: Front Pain Res (Lausanne). 2025 Dec 10;6:1709275. doi: 10.3389/fpain.2025.1709275 (PMC12728057; doi:10.3389/fpain.2025.1709275)
Supplement: Supplementary file 2 [file Datasheet2.pdf]

Appendix 2 – Central sensitization

Response to mechanical temporal summation (RMTS) (§ 4.1.3)

Principle/Aim

- To evaluate the spinal wind-up facilitation process.
- To measure the spinal hyperexcitability.

Equipment

- Meshed cage with litter

- Meshed cage with cushion
- Comfortable bed

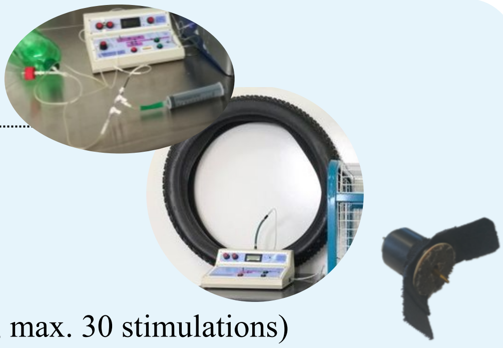

- Temporal summation system (2N, max. 30 stimulations)
- Round-ended metallic pin (2.5 mm Ø, 10 mm long) in an actuator cuff

Acclimatization (< 15 mins. per session)

Days	1	4	7	10	14
	Cage/cushion<br>Look at/sniff device<br>Cuff at the tail's cranial base	Connect cuff to the temporal summation device	Listen to the device's stimulation noise	Apply stimulation with light pressure (1N)	Apply stimulation with light pressure (1N)

Step by step process

Installation

- Turn on the system and set the pressure to 2N
- Place cuff around the tail's cranial base

Basal/relaxed state *

- Place animal in the cage/on the cushion
- Give positive reinforcement (treats, affection)
- Wait few mins.

Take measure *

- Start stimulation
- Observe aversive response (vocalization, agitation, turns repeatedly to the cuff or tries to remove it, licking, jumping or avoidance, *etc.*)
- Stop the system
- Note the number of stimuli

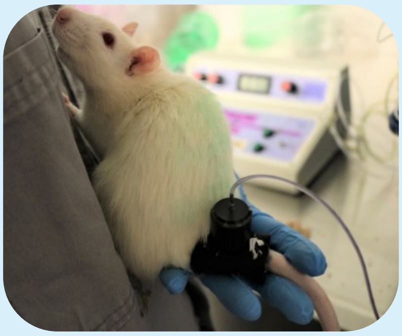

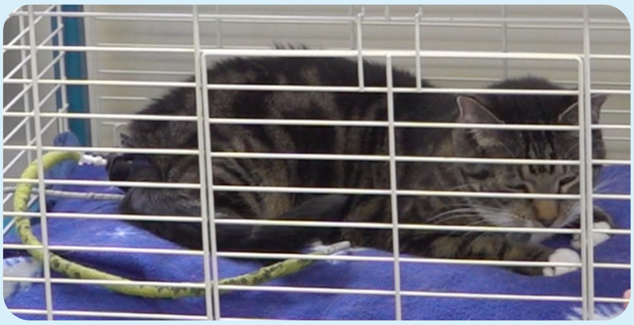

End of the experiment

- Give positive reinforcement
- Remove the animal
- Clean the cage or cushion

Interpretation

↓ Number of tolerated stimuli

Animal **with** central sensitization

* Be careful, read before any assessment ⚠

- If a reflex movement occurs during the first 3 stimulations or when the animal reacts to the noise, this is not a valid response, take another measurement once the animal has returned to its basal state.
- No environmental distraction (noise, treat, light, *etc.*).

RMTS using the Top Cat System from Top Cat Metrology®, Bespoke Measurement Systems, Cambridgeshire, UK
