# Supplementary material for: Translational validity of quantitative sensory testing in chronic pain neuro-sensitization: guide of use and interpretation in osteoarthritis animal models
Source: Front Pain Res (Lausanne). 2025 Dec 10;6:1709275. doi: 10.3389/fpain.2025.1709275 (PMC12728057; doi:10.3389/fpain.2025.1709275)
Supplement: Supplementary file 3 [file Datasheet3.pdf]

Appendix 3 – Pain endogenous inhibition

Conditioned pain modulation (CPM) (§ 4.2.1)

Université de Montréal
GREPAQ

Principle/Aim

To evaluate the endogenous inhibitory control of pain: the diffuse noxious inhibitory control (DNIC) by using the CPM.

Equipment

- Meshed cage, cushion, comfortable bed
- Electronic von Frey® esthesiometer (max. 200g)
- Wagner® algometer (max. 10N)
- Conditioning stimulus (CS):

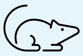

Ear clip positioned with tape for 1 min.

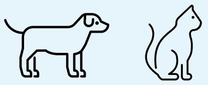

Manometric cuff positioned for 2 mins. on a thoracic limb and inflated at 200 mmHg

Acclimatization

Days	1	4	7	10	14
	Place CS device for a few sec. without activating it	Place the animal in the cage or in contention with the CS device	Measure PWT/PPT with the CS device	Measure PWT/PPT with **light** CS (100 mmHg)	Measure PWT/PPT with **full** CS (200 mmHg)

Step by step process

Basal/relaxed state →
- Place animal in the cage/on the cushion
- Wait 1 min. (rat) or 2 mins. (cat/dog)

Device application * →
- Perpendicular application with device (paw, pad or metatarsus)
- Gradual increase

Take PWT/PPT measure pre-CS * (repeat)
- Observe aversive response (paw withdrawal, vocalization, *etc.*)
- Remove the device
- Note the value

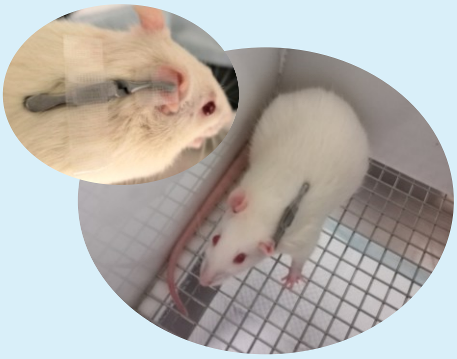

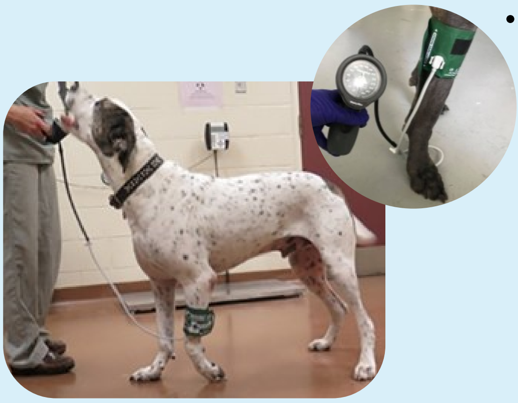

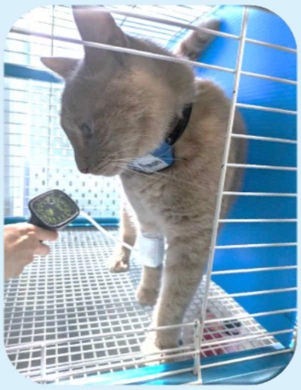

Perform CS
Wait 1 min.

Take PWT/PPT measure post-CS * (repeat)

End of the experiment
- Give positive reinforcement (treats, affection)
- Remove the animal
- Clean the cage or cushion

Interpretation

PWT/PPT post-CS > PWT/PPT pre-CS → Animal with **functional** CPM

PWT/PPT post-CS < PWT/PPT pre-CS → Animal with **non-functional** CPM

* Be careful, read before any assessment ⚠
- The animal MUST stand on its 4 limbs.
- If a reflex movement occurs when the device touches the skin this is not a valid threshold, take another measure once the animal has returned to its basal state.
- No environmental distraction (noise, treat, light, *etc.*).

Electronic von Frey esthesiometer® from IITC Life Sciences Inc., Woodland Hills, CA, USA; Wagner Force Gauge M3-2 from Mark-10 Corporation, Copiague, NY, USA
