# Supplementary material for: Translational validity of quantitative sensory testing in chronic pain neuro-sensitization: guide of use and interpretation in osteoarthritis animal models
Source: Front Pain Res (Lausanne). 2025 Dec 10;6:1709275. doi: 10.3389/fpain.2025.1709275 (PMC12728057; doi:10.3389/fpain.2025.1709275)
Supplement: Supplementary file 4 [file Datasheet4.pdf]

Appendix 4 – Pain endogenous facilitation

PWT/PPT pre- and post-RMTS (§ 4.2.2)

Principle/Aim

To evaluate the endogenous facilitatory control of pain: measure spinal hyperexcitability of second-order neurons.

Equipment

- Meshed cage, cushion, comfortable bed
- Electronic von Frey® esthesiometer (max. 200g)
- Wagner® algometer (max. 10N)
- Temporal summation system (2N, max. 30 stimulations)
- Round-ended metallic pin (2.5 mm Ø, 10 mm long) in an actuator cuff

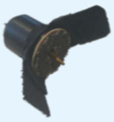

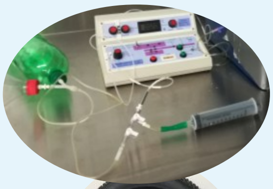

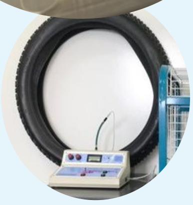

Acclimatization

1 — 4 — 7 — 10 — 14 → Days

Follow the peripheral and central sensitization protocols

Step by step process

Installation
- Turn on the system and set the pressure to 2N
- Place cuff around the tail's cranial base

Basal/relaxed state *
- Place animal in the cage/on the cushion
- Give positive reinforcement (treats, affection)
- Wait few mins.

Take PWT/PPT measure pre-RMTS *
- Observe aversive response (paw withdrawal, vocalization, *etc.*)
- Remove the device
- Note the value

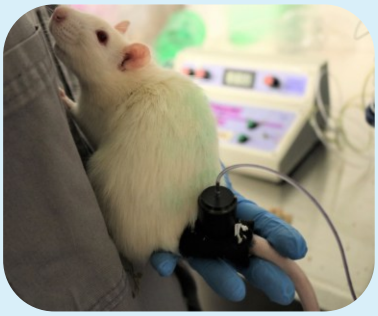

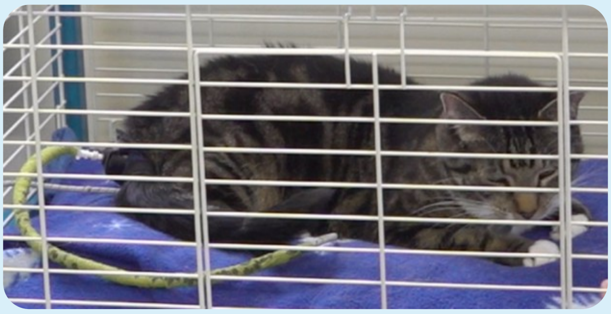

Perform RMTS (see central sensitization protocol)

Take PWT/PPT measure post-RMTS *

< 1 min. after RMTS completed

End of the experiment
- Give positive reinforcement
- Remove the animal
- Clean the cage or cushion

Interpretation

PWT/PPT post-RMTS < PWT/PPT pre-RMTS → Animal with **active** facilitation control

PWT/PPT post-CS > PWT/PPT pre-RMTS → Animal with **counteracted** facilitation

* Be careful, read before any assessment ⚠

- The animal MUST stand on its 4 limbs.
- If a reflex movement occurs during the first 3 stimulations or when the animal reacts to the tool touch or noise, this is not a valid response, take another measurement once the animal has returned to its basal state.
- No environmental distraction (noise, treat, light, *etc.*).

RMTS using the Top Cat System from Top Cat Metrology®, Bespoke Measurement Systems, Cambridgeshire, UK; Electronic von Frey esthesiometer® from IITC Life Sciences Inc., Woodland Hills, CA, USA; Wagner Force Gauge M3-2 from Mark-10 Corporation, Copiague, NY, USA
