# Supplementary material for: Translational validity of quantitative sensory testing in chronic pain neuro-sensitization: guide of use and interpretation in osteoarthritis animal models
Source: Front Pain Res (Lausanne). 2025 Dec 10;6:1709275. doi: 10.3389/fpain.2025.1709275 (PMC12728057; doi:10.3389/fpain.2025.1709275)
Supplement: Supplementary file 5 [file Table1.docx]

Supplementary Material

**Table S1** – Search strategies used to complete Table 2 (quantitative sensory testing validation) and Table 3 (osteoarthritis pain management) with associated references.

PubMed served as the primary database, with Google Scholar consulted when additional references were required. Included references were selected based on: (i) systematic reviews or meta-analyses, (ii) original methodological validation studies, and (iii) efficient reference management, reusing the same sources between Tables 2 and 3 whenever possible. Search pathways for Table 3 were incorporated into those developed for Table 2. The use of asterisks in keyword strings allowed automatic retrieval of all derived forms of a term from its root.

|  | Path used | Number of recorded references |
| --- | --- | --- |
| Table 2 – Quantitative sensory testing to assess osteoarthritis pain mechanisms | | |
| Humans | (human OR people) AND (osteoarth*) AND ("quantitative sensory testing" OR "temporal summation" OR "conditioned pain modulation") | *n* = 257 |
| Rats | (rat OR sprague-dawley OR wistar OR lewis) AND (osteoarth*) AND ("quantitative sensory testing" OR sensiti* OR pain threshold OR hyperalgesi* OR allodyni* OR summation OR wind-up OR hyperexcita* OR "conditioned pain modulation" OR "diffuse noxious inhibitory controls" OR dnic OR descending) | *n* = 683 |
| Cats | (feline OR cat) AND (osteoarth*) AND ("quantitative sensory testing" OR sensiti* OR pain threshold OR summation OR wind-up OR hyperexcita* OR "conditioned pain modulation" OR "diffuse noxious inhibitory controls" OR dnic) | *n* = 44 |
| Dogs | (canine OR dog) AND (osteoarth*) AND ("quantitative sensory testing" OR sensiti* OR pain threshold OR summation OR wind-up OR hyperexcita* OR "conditioned pain modulation" OR "diffuse noxious inhibitory controls" OR dnic) | *n* = 148 |
| Table 3 – Improving osteoarthritis pain management through quantitative sensory testing sensitivity | | |
| Non-steroidal anti-inflammatory drugs (NSAIDs) | non-steroidal anti-inflammatory drugs OR nsaid OR meloxicam OR carprofen OR coxib OR naproxen OR loxoprofen OR ketoprofen OR aspirin OR diclofenac OR ibuprofen | Humans (*n* = 9)  Rats (*n* = 86)  Cats (*n* = 4)  Dogs (*n* = 15) |
| Corticosteroids | corticosteroid OR methylprednisolone | Humans (*n* = 4)  Rats (*n* = 3)  Cats (*n* = 0)  Dogs (*n* = 2) |
| Anti-nerve growth factor (NGF) monoclonal antibodies | anti-nerve growth factor OR anti-ngf OR monoclonal antibodies OR monoclonal antibody OR bedinvetmab OR frunevetmab OR fasinumab OR tanezumab | Humans (*n* = 1)  Rats (*n* = 9)  Cats (*n* = 0)  Dogs (*n* = 3) |
| Opioids | opioid OR tramadol OR morphine OR oxycodone OR tapentadol OR hydromorphone | Humans (*n* = 7)  Rats (*n* = 37)  Cats (*n* = 4)  Dogs (*n* = 0) |
| Gabapentinoids | gabapentinoid OR gabapentin OR pregabalin | Humans (*n* = 2)  Rats (*n* = 21)  Cats (*n* = 2)  Dogs (*n* = 1) |
| Acetaminophen | acetaminophen OR paracetamol | Humans (*n* = 4)  Rats (*n* = 5)  Cats (*n* = 0)  Dogs (*n* = 1) |
| Selective serotonin/norepinephrine reuptake inhibitors and tricyclic antidepressants | selective serotonin reuptake inhibitors OR ssri OR serotonin-norepinephrine reuptake inhibitors OR snri OR tricyclic antidepressants OR duloxetine OR fluoxetine OR sertraline OR citalopram OR venlafaxine OR vortioxetine OR amitriptyline OR clomipramine OR milnacipran | Humans (*n* = 5)  Rats (*n* = 9)  Cats (*n* = 0)  Dogs (*n* = 1) |
| Cannabinoids | cannabinoid OR endocannabinoid OR cannabidiol OR cb1 OR cb2 OR dronabinol | Humans (*n* = 1)  Rats (*n* = 22)  Cats (*n* = 0)  Dogs (*n* = 1) |
| Arthroplasty | arthroplasty OR joint replacement | Humans (*n* = 58)  Rats (*n* = 15)  Cats (*n* = 6)  Dogs (*n* = 3) |
